# Accelerating haploid induction rate and haploid validation through marker-assisted selection for *qhir1* and *qhir8* in maize

**DOI:** 10.3389/fpls.2024.1337463

**Published:** 2024-03-05

**Authors:** Kanogporn Khammona, Abil Dermail, Khundej Suriharn, Thomas Lübberstedt, Samart Wanchana, Burin Thunnom, Wasin Poncheewin, Theerayut Toojinda, Vinitchan Ruanjaichon, Siwaret Arikit

**Affiliations:** ^1^ Department of Agronomy, Faculty of Agriculture at Kamphaeng Saen, Kasetsart University, Nakhon Pathom, Thailand; ^2^ Department of Agronomy, Faculty of Agriculture, Khon Kaen University, Khon Kaen, Thailand; ^3^ Plant Breeding Research Center for Sustainable Agriculture, Faculty of Agriculture, Khon Kaen University, Khon Kaen, Thailand; ^4^ Department of Agronomy, Iowa State University, Ames, IA, United States; ^5^ National Center for Genetic Engineering and Biotechnology (BIOTEC), National Science and Technology Development Agency (NSTDA), Pathum Thani, Thailand; ^6^ Rice Science Center, Kasetsart University, Nakhon Pathom, Thailand

**Keywords:** hybrid breeding, doubled haploid, haploid induction, haploid identification, molecular assay

## Abstract

Doubled haploid (DH) technology becomes more routinely applied in maize hybrid breeding. However, some issues in haploid induction and identification persist, requiring resolution to optimize DH production. Our objective was to implement simultaneous marker-assisted selection (MAS) for *qhir1* (*MTL/ZmPLA1/NLD*) and *qhir8* (*ZmDMP*) using TaqMan assay in F_2_ generation of four BHI306-derived tropical × temperate inducer families. We also aimed to assess their haploid induction rate (HIR) in the F_3_ generation as a phenotypic response to MAS. We highlighted remarkable increases in HIR of each inducer family. Genotypes carrying *qhir1* and *qhir8* exhibited 1 – 3-fold higher haploid frequency than those carrying only *qhir1*. Additionally, the *qhir1* marker was employed for verifying putative haploid seedlings at 7 days after planting. Flow cytometric analysis served as the gold standard test to assess the accuracy of the *R1-nj* and the *qhir1* marker. The *qhir1* marker showed high accuracy and may be integrated in multiple haploid identifications at early seedling stage succeeding pre-haploid sorting via *R1-nj* marker.

## Introduction

Maize is one of the most important cereal crops in the world as food, feed, and fuel (Prasanna, 2012). The success of hybrid maize breeding relies on robust pipelines of germplasm, genetics, phenotyping, and selection processes (Cooper et al., 2014). Traditionally, the breeding process for the market release of a new cultivar extended over a decade, until the advent of doubled haploid (DH) technology (Chaikam et al., 2019). A notable advantage of DH technology is associated with the substantial reduction of breeding cycles required to develop fully homozygous lines within just two generations (Geiger and Gordillo, 2009). Haploids can be produced *in vitro* or *in vivo*. The *in vitro* method requires laboratory procedures, where gametophytic tissues such as microspores and egg cells are used to produce paternal and maternal haploids, respectively. However, this method gains low success rates due to the high levels of genotype dependency (Jacquier et al., 2021). The *in vivo* method involves four main steps: (1) haploid induction, (2) haploid identification, (3) haploid genome doubling, and (4) self-pollination of haploid plants to obtain DH_0_ seeds (Chaikam et al., 2019). For maternal haploid induction, haploid inducers are used as male parents to pollinate source germplasm for haploid induction. Efficient DH line production depends on the availability of inducer genotypes with high induction ability.

In 2012, a QTL study involving four populations, all sharing the inbred inducer UH400 as common parent, identified 8 QTL. Notably, *qhir1* and *qhir8* emerged as two major QTL located on chromosomes 1 and 9, explaining 66% and 20% of the genetic variance, respectively (Prigge et al., 2012). The *qhir1* region in bin 1.04 plays pivotal roles in triggering haploid induction, gametophytic segregation distortion, and embryo abortion (Barret et al., 2008; Prigge et al., 2012; Xu et al., 2013). Mutation of the gene *MTL/ZmPLA1/NLD* in *qhir1* has been shown to generate an average haploid induction rate (HIR) up to 6.7% (Gilles et al., 2017; Kelliher et al., 2017; Liu et al., 2017). However, *qhir1* is not sufficient for commercial productions of DH lines. To fully leverage this technology, the average HIR of modern haploid inducers should surpass 10% (Hu et al., 2016). Zhong et al. (2019) discovered a novel gene named *ZmDMP* underlying QTL *qhir8*. A mutation of *ZmDMP* markedly enhances haploid induction, resulting in a 2–3-fold increase in HIR. It is important to note that both *MTL/ZmPLA1/NLD* and *ZmDMP* act synergistically, suggesting the potential for a substantial 5–6-fold increase in the HIR when both mutations are present (Zhong et al., 2019). Marker assisted selection (MAS) for *qhir1* has been applied to improve the HIR of maternal haploid inducers in different maize backgrounds. For instance, Chaikam et al. (2018) were able to obtain promising second-generation Tropically Adapted Inducer Lines (2GTAILs) with an average HIR of 13.1%, a 48.9% improvement over TAILs. Liu et al. (2022) developed an elite oil haploid inducer, CHOI4, with an averaged HIR of 15.8%, a 58.0% increase compared to CAU2, the founder parent of CHOI4. While these results are promising, further enhancements could be achieved through MAS for two loci*, qhir1* and *qhir8*. Considering that HIR is a polygenic trait, selection of a single locus may not be sufficient to obtain inducers with optimum HIR (Dong et al., 2014). Nevertheless, limited evidence exists to illustrate the feasibility of MAS for both loci simultaneously in breeding haploid inducers for high HIR.

Haploids are commonly identified via the *R1-navajo* (*R1-nj*), a dominant monogenic biomarker (Nanda and Chase, 1966) integrated in haploid inducers. This marker distinguishes progeny seeds derived from haploid induction based on anthocyanin expression in different parts of the kernel. Haploid kernels show a purple crown in the endosperm but a colorless scutellum in the embryo, while diploids express both purple endosperm and embryo (Dermail et al., 2021). Despite practical and non-destructive features, the effectiveness of *R1-nj* expressions may be constrained by the presence of dominant *C1* anthocyanin inhibitors (Chaikam et al., 2015), naturally occurring anthocyanins in donor germplasm (Chaikam et al., 2016), morpho-physiological kernel properties (Prigge et al., 2011; Trentin et al., 2022), and environments (Sintanaparadee et al., 2022; Dermail et al., 2023; Thawarorit et al., 2023). These factors contribute to high misclassification rates (MCRs), hindering selection gains on HIR and emphasizing the need for alternative markers for haploid selection. While simple sequence repeat (SSR) has been successfully used in maize haploid identification (Qiu et al., 2014; Dong et al., 2018; Li et al., 2021), the practical use of SNP markers for that purpose is still lacking. Since most paternal chromosomes of inducers are excluded from haploid embryonic cells within a week after pollination, the haploid individuals carry only maternal chromosomes from the donor germplasm (Zhao et al., 2013). Codominant SNP markers can differentiate between homozygotes (donor female) and heterozygotes (F_1_ diploids). Considering remarkable allelic variation for *qhir1* and *qhir8* haploid inducers versus non-inducer genotypes, there is an encouraging prospect of applying these loci for haploid identification using TaqMan probes. Kelliher et al. (2017) employed TaqMan assays for *qhir1*, proposing that haploids carry zero copies of the *mtl* allele and two copies of the maternal *MTL* allele, whereas diploids carry one copy of the *mtl* allele and one copy of the *MTL* allele.

Our study aimed to utilize the *qhir1* and *qhir8* loci in marker-assisted selection, with a dual focus on breeding haploid inducers for high HIR and accurately identifying true haploids in maize. We hypothesized that (i) inducer genotypes carrying *qhir1* and *qhir8* should demonstrate a higher capacity to induce haploids compared to those carrying *qhir1* alone and (ii) molecular markers using TaqMan assay are more reliable than the *R1-nj* marker when validated with flow cytometry. This study will provide an insight into the advantages of molecular assays, especially TaqMan probes, to accelerate the improvements of haploid inducers underpinning HIR. Additionally, it seeks to enhance the accuracy of identifying true haploids at early seedling stage.

## Materials and methods

### Breeding scheme, haploid induction, and HIR evaluation

A temperate inbred inducer, BHI306, and four tropical inducer families (K8, K11, KHI49, and KHI54) were selected as founder parents. The BHI306 genotype, an RWS/RWK-76-derived haploid inducer, has 10–15% of HIR, carries both *qhir1* and *qhir8* loci (**Supplementary Table S1** and **Supplementary Figure S1**), kernel anthocyanin *R1-nj* and red root *Pl-1* selectable markers. BHI306 was developed by the DH Facility of Iowa State University (DHF-ISU) (https://www.doubledhaploid.biotech.iastate.edu/). Four genotypes, K8, K11, KHI49, and KHI54 belong to qhir1−/qhir8− group (**Supplementary Table S1** and **Supplementary Figure S1**), Stock-6-derived haploid inducers, had low HIRs (<6.0%) but possess favorable tropical adaptations. These genotypes were developed by the Plant Breeding Research Center for Sustainable Agriculture of Khon Kaen University in Thailand (Dermail et al., 2021; Sintanaparadee et al., 2022; Thawarorit et al., 2023). A 1 × 4 factorial mating scheme was performed by assigning BHI306 as a male and four tropical inducers as females to establish four tropical × temperate inducer base populations including K8/BHI306, K11/BHI306, KHI49/BHI306, and KHI54/BHI306. In the F_2_ generation, approximately 100 F_2_ seedlings per inducer population underwent random marker-assisted selection (MAS) for *qhir1* and *qhir8*. Plants carrying *qhir1* only and both *qhir1* and *qhir8* were labeled as *qhir1+/qhir8−* and *qhir1+/qhir8+* genotypes, respectively. These targeted genotypes were subsequently transplanted into the field and self-pollinated to obtain F_3_ seeds. At that generation, we did not perform haploid induction. Thus, there was no preliminary information regarding the actual HIR. At the F_3_ generation, repeated genotyping of each individual plant and phenotyping on actual HIR were performed in each population (**Supplementary Table S1**).

Maternal haploid induction was performed to evaluate HIRs. A commercial hybrid Pacific789 (P789), developed by Pacific Seeds, Thailand, was used as a donor female. This genotype is resistant to tropical diseases, high-yielding, and large-seeded with flat embryos, facilitating haploid selection based on the *R1-nj* marker at the seed stage. Each F_3_ inducer plant in each *qhir* genotype and family was used to pollinate four donor ears to minimize the errors due to unstable inducer pollen. Shoot bagging and detasseling of donor plants were routinely performed to prevent pollen contamination.

Haploid seed was selected via the *R1-nj* marker at the seed stage. Haploids showed a purple crown endosperm but a colorless embryo, while diploids expressed purple colorations on both crown endosperm and embryo (Nanda and Chase, 1966; Dermail et al., 2023). The HIR was calculated as the frequency of haploid seeds per induction cross, as follows:


HIR (%) =seed number of putative haploidseed set x 100

where seed set represents the total seed number of haploid seeds, diploid seeds, and the seeds without the *R1-nj* marker.

About 10 putative haploid seeds per genotype in each inducer family were sampled for further true haploid confirmation through molecular assays.

### Marker development

**Figure 1:**
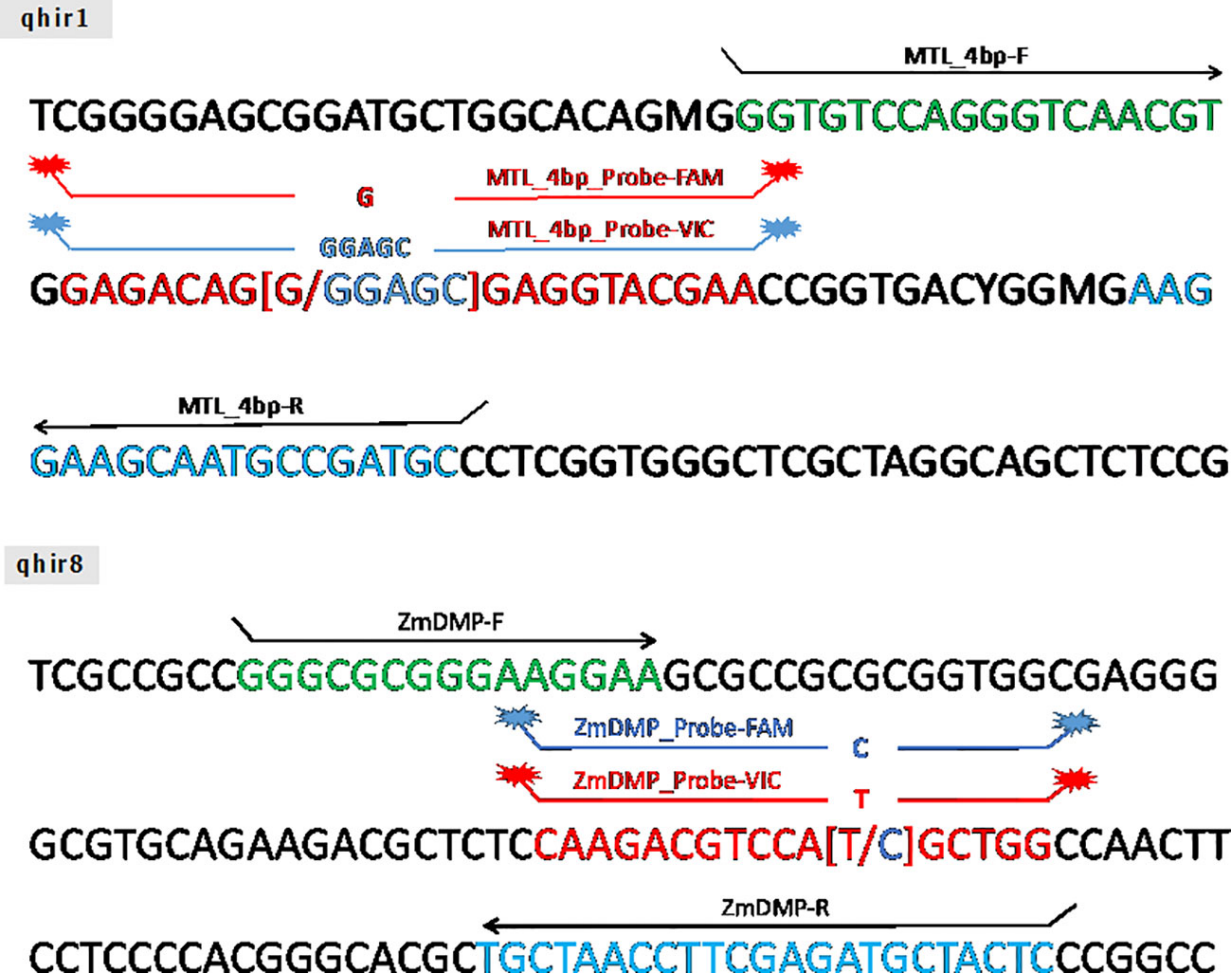
The schematic of TaqMan^®^ probe design on *MATRILINEAL* gene (GRMZM2G471240) and *ZmDMP* gene (GRMZM2G465053).

Two TaqMan^®^ markers (*qhir1* and *qhir8*) for two targeted genes namely *MATRILINEAL* (*MTL/ZmPLA1/NLD*) and *ZmDMP*, respectively, were constructed (**Figure 1**). The marker for the *MTL* gene (GRMZM2G471240) was developed at 4 bp (CGAG) insertion in the 4th exon of the gene that led to premature stop codon (Gilles et al., 2017; Kelliher et al., 2017; Liu et al., 2017). The *ZmDMP* gene (GRMZM2G465053) was developed at single nucleotide substitution from T to C at 131 bp on coding sequence that led to amino acid change from methionine to threonine (Zhong et al., 2019).

### Genotyping and DNA extraction

High-quality genomic DNA (gDNA) was isolated from maize leaves at 14 days after germination using the DNeasy^®^ Plant Mini Kit (QIAGEN, Germany). Genotyping for *qhir1* and *qhir8* markers was carried out with ready-to-order TaqMan assays (Thermo Fisher Scientific, Watham, MA USA) (**Figure 1**). In the amplification process, 20 ng gDNA was utilized. For the PCR reaction, the total volume was 5 µl composed of 2 µl of template DNA, 1.5 µl of 2X TaqMan^®^ Gene Expression Master Mix (Thermo Fisher Scientific, Watham, MA USA), 0.0375 µl of TaqMan assay, and 1.4625 µl of dH2O. The PCR cycling conditions were set at 95°C for 5 min, followed by 36 cycles at 94°C for 30 s, 60°C for 1 min, and 60°C for 2 min. For the PCR product, the amplicons were melted at 60°C using QuantStudio 6 Real-Time PCR Systems (Thermo Fisher Scientific, Watham, MA USA) for 30 s to detect single nucleotide polymorphism (SNP).

### Flow cytometry analysis

Three subsets of populations derived from induction crosses between female donor P789 and three male inducers, BHI306, KHI49/BHI306, and KHI54/BHI306, were used for haploid validation via flow cytometry analysis. The number of samples was 24, derived from false positives previously assumed as putative haploids based on the *R1-nj* marker but eventually true diploids regarding the *qhir1* marker. Those 24 samples composed of 1 putative haploid of P789/BHI306, 10 putative haploids of P789/(KHI49/BHI306), and 13 putative haploids of P789/(KHI54/BHI306). The FC analysis on those 24 samples served as the gold standard classification method to verify if *qhir1* marker is reliable to determine the true haploids. The FC graph of each sample can be found in the **Supplementary Figure S3**, and the result of FC analysis corresponding to the *qhir1* marker assay can be found in **Table 1**.

**Table 1:** Haploid validation via *qhir1* marker and flow cytometry (FC) analysis of 24 false positives derived from subsets of F_1_ induction crosses between female donor P789 and three male inducers BHI306, KHI49/BHI306, and KHI54/BHI306.

No.	Sample name	*qhir1*	FC	*qhir1* vs. FC	
				R^2^	*p-value*
1	P789/BHI306-F_1__n-5	2n	2n	1.00	2.2E−16
2	P789/(KHI49/BHI-F_3_)-F_1__n-1-2-6	2n	2n		
3	P789/(KHI49/BHI-F_3_)-F_1__n-1-3-2	2n	2n		
4	P789/(KHI49/BHI-F_3_)-F_1__n-1-4-8	2n	2n		
5	P789/(KHI49/BHI-F_3_)-F_1__n-1-6-5	2n	2n		
6	P789/(KHI49/BHI-F_3_)-F_1__n-1-7-1	2n	2n		
7	P789/(KHI49/BHI-F_3_)-F_1__n-1-14-7	2n	2n		
8	P789/(KHI49/BHI-F_3_)-F_1__n-1-15-3	2n	2n		
9	P789/(KHI49/BHI-F_3_)-F_1__n-1-20-10	2n	2n		
10	P789/(KHI49/BHI-F_3_)-F_1__n-2-2-6	2n	2n		
11	P789/(KHI49/BHI-F_3_)-F_1__n-2-2-9	2n	2n		
12	P789/(KHI54/BHI-F_3_)-F_1__n-1-1-3	2n	2n		
13	P789/(KHI54/BHI-F_3_)-F_1__n-1-1-6	2n	2n		
14	P789/(KHI54/BHI-F_3_)-F_1__n-1-2-3	2n	2n		
15	P789/(KHI54/BHI-F_3_)-F_1__n-1-3-4	2n	2n		
16	P789/(KHI54/BHI-F_3_)-F_1__n-1-4-3	2n	2n		
17	P789/(KHI54/BHI-F_3_)-F_1__n-1-10-1	2n	2n		
18	P789/(KHI54/BHI-F_3_)-F_1__n-1-11-1	2n	2n		
19	P789/(KHI54/BHI-F_3_)-F_1__n-1-12-7	2n	2n		
20	P789/(KHI54/BHI-F_3_)-F_1__n-1-16-2	2n	2n		
21	P789/(KHI54/BHI-F_3_)-F_1__n-1-17-1	2n	2n		
22	P789/(KHI54/BHI-F_3_)-F_1__n-1-18-1	2n	2n		
23	P789/(KHI54/BHI-F_3_)-F_1__n-1-19-2	2n	2n		
24	P789/(KHI54/BHI-F_3_)-F_1__n-2-3-10	2n	2n		

R^2^ coefficient of determination.

All 24 false positives were previously classified as putative haploids based on the *R1-nj* marker, but then they were verified as true haploids based on the *qhir1* marker. The result of the FC graph on each of the 24 samples can be found in **Supplementary Figure S3**.

Two maize leaves at 14 days after germination were cut about 3 cm in length (50-100 mg fresh weight) and placed into a plastic petri dish on ice. Then, 1.5 ml of LB01 buffer (15 mM Tris, 2 mM Na2EDTA, 0.5 mM spermine.4HCl, 80 mM KCl, 20 mM NaCl, 0.1% (v/v) Triton X-100, pH 7.5) (Doležel, 1997) was added, and the leaves were chopped in this buffer using a razor blade to facilitate the release of the nuclei (Pfosser et al., 1995). After that, 500 ul of the cell solution was transferred into a 1.5 ml tube. Propidium Iodide 1 mg/mlI-stained nuclei and RNaseA were then added to the solution. The BD Accuri™ C6 Plus flow cytometer (BD Biosciences, USA) was employed for measurement. The ploidy status of each sample can be determined by the fluorescence intensity of stained cell nuclei isolated from plant tissue. The peak value (G1) of haploid is commonly set to half of the diploid reference (**Supplementary Figure S3**).

### Statistical analysis

A total of 237 inducer plants were evaluated for HIR performance including K8/BHI306 (32 plants), K11/BHI306 (54 plants), KHI49/BHI306 (52 plants), and KHI54/BHI306 (99 plants). Each induced donor ear was represented as a technical replicate, resulting in four replications for each inducer plant within each genotype and family. The HIR for each genotype was calculated as the mean HIR across these four replications. The data were subjected to the unpaired samples t-test with 95% confidence interval (CI), Tukey’s Honestly Significant Difference (HSD) Test at 5%, and linear regression analysis.

## Results

### Haploid inducer breeding via marker-assisted selection for *qhir1* and *qhir8*

**Figure 2:**
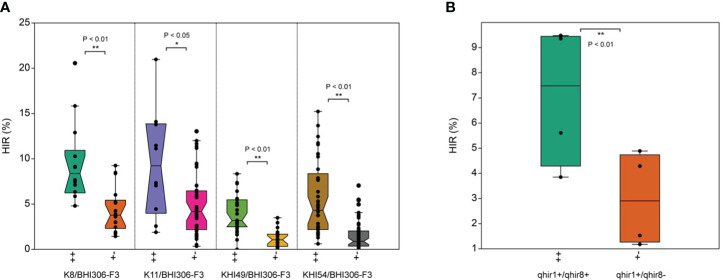
Haploid induction rate (HIR) in F_3_ inducer families with *qhir1+/qhir8+* (++) and *qhir1+/qhir8−* (+−) genotypes involving **(A)** four different inducer parents; and **(B)** averaged means over four respective inducer populations. *, and ** data significant through paired samples t-test at *P*<0.05 and *P*<0.01, respectively.

The median HIR of *qhir1+/qhir8+* genotypes was significantly (*P*<0.01) higher than that of the *qhir1+/qhir8−* genotypes within each F_3_ inducer family (**Figure 2A**). Across the four families, the average HIR for the *qhir1+/qhir8+* genotype ranged from 3.85 to 9.48%, while the average HIR for the *qhir1+/qhir8−* genotype was significantly (*P*<0.01) lower, ranging from 1.18 to 4.89% (**Figure 2B**; **Table 2**). This suggests that inducer genotypes fixed for both targeted loci for HIR, *qhir1* and *qhir8*, have remarkable abilities to induce haploids, showing an increase of 3–5% or 1–3-fold higher than inducer genotypes fixed for *qhir1* only.

**Table 2:** The mean comparison between *qhir1+/qhir8+* and *qhir1+/qhir8−* genotypes of each F_3_ family on haploid induction rate (HIR, %).

Population name	Gene combination	HIR (%)	PNU	TPN	*p*-value	PVE (%)
K8/BHI306-F_3_	*qhir1+/qhir8+*	9.36 ^A^	14	32	1.05E−03 **	36
	*qhir1+/qhir8−*	4.29 ^a^	18			
K11/BHI306-F_3_	*qhir1+/qhir8+*	9.48 ^A^	10	54	4.01E−02 *	17
	*qhir1+/qhir8−*	4.89 ^a^	44			
KHI-49/BHI306-F_3_	*qhir1+/qhir8+*	3.85 ^B^	31	52	6.21E−08 **	39
	*qhir1+/qhir8−*	1.18 ^b^	21			
KHI-54/BHI306-F_3_	*qhir1+/qhir8+*	5.61^B^	41	99	1.91E−07 **	33
	*qhir1+/qhir8−*	1.53 ^b^	58			

PNU the number of plants; TPN the total number of plants; PVE proportion of variance in phenotypes explained (%).

HIR means (%) followed by different uppercase letters within the same *qhir1+/qhir8+* genotype are significantly different based on Tukey’s Honestly Significant Difference (HSD) Test at 5%.

HIR means (%) followed by different lowercase letters within the same *qhir1+/qhir8−* genotype are significantly different based on Tukey’s Honestly Significant Difference (HSD) Test at 5%.

* and ** data significant through paired samples t-test at *P*<0.05 and *P*<0.01, respectively.

The proportion of phenotypic variation explained (PVE) across inducer families ranged from 17% to 39% (**Table 2**). These values, within acceptable ranges, indicated that MAS for two loci was effective in identifying haploid inducers with high HIR. We also found that the HIR between families within the same *qhir1+/qhir8+* genotype was significantly different (**Table 2**). For instance, families K8/BHI306 and K11/BHI306 demonstrated a significantly higher HIR than families KHI-49/BHI306 and KHI-54/BHI306. The evidence of low %PVE (<50%) (**Table 2**), outliers, and overlapping values between two inducer groups on HIR (**Figure 2**), suggests the potential existence of other minor QTL influencing HIR.

### Haploid validation via *qhir1* marker and flow cytometry analysis

**Figure 3:**
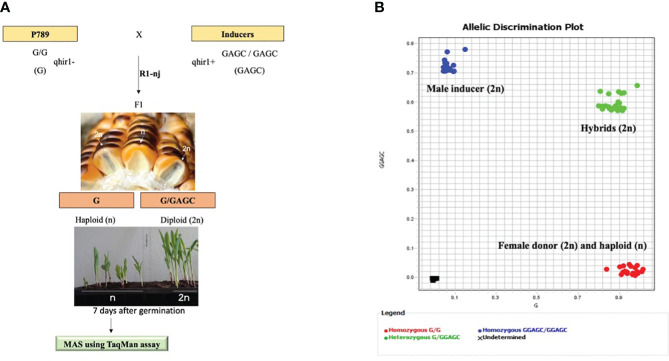
Workflow for haploid identification through the *R1-nj* and haploid validation by MAS using *qhir1* TaqMan assay **(A)** and the allelic distribution plot from TaqMan assay **(B)**. The diploid and haploid classes are represented by the green and red dots, respectively. Genotype BHI306 was used as male inducer, whereas genotype P789 was used as female donor. The SNP graphs for other populations can be found in **Supplementary Figure S2**.

Marker-assisted selection (MAS) for *qhir1* was applied to validate putative haploids and diploids derived from the *R1-nj* marker system as a preliminary haploid identification among the F_1_ progenies of induction crosses. Both parents, BHI306 and P789, were included as positive and negative controls for *qhir1*, respectively (**Figure 3**). Through the TaqMan assay, all samples of P789, the female donor, were found to be homozygous for *qhir1−* (G/G), while all samples of BHI306, the male inducer, were homozygous *qhir1+* (GGAGC/GGAGC). The sample progenies were then distributed into two pools according to haplotypes: (1) the diploid class, heterozygous for *qhir1* (G/GGAGC) and (2) the haploid class, homozygous for *qhir1−* (G/G), which was grouped with the donor female P789 (**Figure 3B**, **Table 3**). Similar results for other populations can be seen in **Supplementary Table S2** and **Supplementary Figure S2**. A few numbers of false positives were found in putative haploid populations derived from induction crosses, accounting for 1, 10, and 13 samples in populations P789/BHI306-F_1_, P789/(KHI49/BHI306-F_3_)-F_1_, and P789/(KHI54/BHI306-F_3_)-F_1_, respectively (**Table 3**). The reliability of the *qhir1* for haploid determination was further validated by flow cytometric analysis. We found that the result of FC analysis among 24 false positives (**Supplementary Figure S3**) corresponded to the *qhir1* marker, as indicated by R^2 ^= 1.00 (**Table 1**). This implies that the *qhir1* marker using the TaqMan assay was effective to identify true haploids, indicated by a 0-false positive rate, which could thus serve as an alternative gold standard test compared to flow cytometry in future. It also suggests that a single SNP marker, like *qhir1*, is ultimately sufficient for haploid identification to reduce the cost of genotyping.

**Table 3:** Ploidy identification (haploid vs. diploid) via TaqMan assay for *qhir1* in a sub-set population of induction crosses between a male inducer BHI306 and a female donor P789.

No	Population name	*qhir1+* (GGAGC/GGAGC)	*qhir1+/qhir1−* (GGAGC/G)	*qhir1−* (G/G)	Total
1	BHI306 – male inducer	10	0	0	10
2	KHI49/BHI306-F_3_ – male inducer	31	0	0	31
3	KHI54/BHI306-F_3_ – male inducer	41	0	0	41
4	P789 – female donor	0	0	7	7
5	P789/BHI306-F_1_ – putative haploid	0	1	10	11
6	P789/BHI306-F_1_ – putative diploid	0	10	0	10
7	P789/(KHI49/BHI306-F_3_)-F_1_ – putative haploid	0	10	146	156
8	P789/(KHI49/BHI306-F_3_)-F_1_ – putative diploid	0	27	0	27
9	P789/(KHI54/BHI306-F_3_)-F_1_ – putative haploid	0	13	162	175
10	P789/(KHI54/BHI306-F_3_)-F_1_ – putative diploid	0	28	0	28
Total		82	89	325	496

Plant samples with *qhir1+/qhir1−* are defined as true diploids.

Plant samples with *qhir1−* are defined as true haploids.

Putative haploid and diploid are based on the preliminary haploid identification via the *R1-nj* marker.

## Discussion

Marker-assisted selection (MAS) may accelerate breeding programs by indirectly selecting target traits using molecular markers tightly linked to underlying genes (Xu and Crouch, 2008). Plant breeders can benefit from this approach especially when targeted traits pose challenges for improvement through traditional phenotypic selections. Technical issues such as resource intensiveness and genetic properties like low heritability, complex inheritance, and presence of recessive alleles make phenotypic selection difficult (Koebner, 2004; Collard et al., 2005; Xu et al., 2005). It is suitable for our breeding objectives to accelerate the rates of haploid induction (HIR) possessing multiple recessive alleles and QTL (Prigge et al., 2012) and prone to the environments of haploid induction (Kebede et al., 2011; De La Fuente et al., 2018; Sintanaparadee et al., 2022).

The effectiveness of MAS for *qhir1* has been reported in the breeding high-oil inducers (Dong et al., 2014) and the development of CIMMYT second-generation Tropically Adapted Inducer Lines (CIM2GTAILs) (Chaikam et al., 2018). Trentin et al. (2020) suggested a stratified MAS approach, initially targeting the *mtl* allele or *qhir1* in a large F_2_ population and later for *zmdmp* allele or *qhir8* in F_3_ plants carrying the *mtl* allele or *qhir1*. In our study, we validated the efficacy of simultaneous MAS for *qhir1* and *qhir8* in F_2_ segregating populations, leading to enhanced HIR in F_3_ genotypes by 1–3-fold. We also noticed that the genotype of *qhir1−/qhir8+* and heterozygous *qhir1/qhir8+* showed lower HIR than genotypes with *qhir1+* (data not shown). Our findings align with Zhong et al. (2019), who identified a novel mutation in the *ZmDMP* gene in the CAUHOI (*qhir1+*) genotype and demonstrated its impact on HIR. They found that the genotype with qhir1+/qhir8+ exhibited inflating HIR by 5–6-fold compared to *qhir1+/qhir8−*. The implementation of MAS in the early generations proves beneficial by significantly reducing the number of F_3_ plants that need evaluation for actual HIR through resource-intensive haploid induction and haploid selection. Chen et al. (2020) also reported the effectiveness of simultaneous MAS for *qhir1* and *qhir8*, resulting in a substantial increase in HIR by 3–14% and the elimination of approximately 90% of low-HIR genotypes.

Our study did not include inducer families with *qhir8* only because we aimed to investigate the synergistic effects between *qhir1* and *qhir8* on HIR. Previous studies have reported that *qhir8* alone resulted in poor or even null HIR. For instance, Chen et al. (2020) reported that the HIR of the plants with *qhir8* only ranged from 0.70% to 1.04%, which was significantly lower than either those that carried a heterozygous *qhir1* allele or those that carried a homozygous *qhir1*, with HIRs of 3.77% to 5.27% and 10.02% to 14.42%, respectively.

Previous studies reported six minor QTL (*qhir2, qhir3, qhir4, qhir5, qhir6, and qhir7*) (Prigge et al., 2012) and a novel gene, *ZmPLD3* (Li et al., 2021). Mutations of the *ZmPLD3* gene resulted in a haploid induction rate (HIR) comparable to that of the homozygous recessive *MTL* gene. This mutation showed synergistic effects rather than functional redundancy in tripling HIR in the presence of the homozygous recessive *MTL* gene. Later in 2022, Meng and colleagues manipulated the Stock6-derived inducer lines by overexpressing maize *CENH3* fused with different fluorescent protein tags and found that the engineered Stock6-derived lines showed a noticeable increase in the maternal HIR up to 16.3%, which was increased by ~6.1% than Stock6-derived lines control (Meng et al., 2022). Hu et al. (2016) found two minor QTL responsible for HIR expression, namely *qhir11* and *qhir12*, which are closely linked to the major QTL *qhir1*. While the *qhir11* was not diagnostic for differentiating inducers and non-inducers, the *qhir12* had a haplotype allele common to all inducer lines observed but not found in all non-inducers studied. In addition, they noticed that the *qhir12* region was related to three candidate genes involved in DNA or amino acid binding. (Nair et al., 2017) performed a genome wide association study (GWAS) and identified more than 20 SNPs associated with HIR in two different association mapping panels. A recent genome-wide association study (GWAS) involving 159 haploid inducers has confirmed the polygenic nature of HIR and identified a major gene near *MTL*, a significant QTL on chromosome 10, and other minor QTL on six of the ten chromosomes (Trentin et al., 2023a). It is conceivable that these QTL, or even undiscovered ones, may be present in our inducer genotypes, highlighting the need for further investigations to discover novel QTL conferring HIR. Drawing insights from Prigge et al. (2012), this endeavor is feasible, as the number of QTL and the magnitude of QTL effects for HIR can vary across populations and generations.

This present study serves as a continuation of the haploid inducer breeding program, focusing on achieving high HIR and local adaptation to the tropical savanna in Thailand. In our previous reports, relying solely on phenotypic selection in the breeding strategy did not yield promising haploid inducers with satisfactory HIR, i.e., below 6% in two families KHI49 and KHI54 (Dermail et al., 2021) and two populations K8 and K11 (Thawarorit et al., 2023). The incorporation of genetic recombination with BHI306, an elite inducer stock carrying favorable alleles for HIR, and the implementation of precise selections such as MAS for simultaneous loci have now enabled us to obtain promising inducer genotypes. Notably, some individual plants within *qhir1+/qhir8+* genotypes in families K8/BHI306 and K11/BHI306 demonstrated HIRs exceeding 20%, surpassing both founder parents (**Supplementary Table S1**).

The significant variations for HIR among families within the same qhir1+/qhir8+ genotype (**Table 2**) imply the importance of the genetic background of founder parents to establish those inducer families. We noticed that families KHI-49/BHI306 and KHI-54/BHI306 had significantly lower abilities to induce haploids than families K8/BHI306 and K11/BHI306. Although the four females (KHI-49, KHI-54, K8, and K11) derived from the same haploid inducer, Stock-6, they experienced different selection schemes. Regarding the pedigree information, the females KHI-49 and KHI-54 had experienced long-term selections, including six for non-HIR related traits and the following three for *R1-nj* kernel marker. Some favorable alleles responsible for HIR may be lost during selections since Chaikam et al. (2019) argued that non-inducer pollen showed selection advantages over inducer pollen. In contrast, the females K8 and K11 only experienced one selection cycle among F_2_ populations derived from crosses between Stock-6 haploid inducer and non-inducer waxy maize germplasm. We assumed that the proportion of HIR-related favorable alleles in the K8 and K11 genotypes was higher than in KHI-49 and KHI-54.

Although *per se* on HIR can be altered by different testing environments and donor germplasm (Kebede et al., 2011; Prigge et al., 2011; De La Fuente et al., 2018; Sintanaparadee et al., 2022), our current finding suggests the presence of transgressive segregants in F_3_ families. We recommend further phenotypic evaluations in inducer families with *qhir1+*/*qhir8+* genotypes, focusing on traits related to the ideotype of haploid inducers, such as plant height, ear height, flowering behaviors, tassel and pollen attributes, and seed set. This assessment will help determine the feasibility of those genotypes in haploid induction stage, whether in induction nurseries or isolation fields (Trentin et al., 2020; Trentin et al., 2023b). Considering the polygenic nature governing HIR and those mentioned agronomic traits, genomic selection approach can be applied to simultaneously identify promising parents to generate progenies with favorable performance on targeted traits prior to field evaluation. Almeida et al. (2020) implemented genomic prediction for cross prediction and parental selection in a haploid inducer breeding program with varying levels of accuracy depending on traits evaluated and suggested that HIR and agronomic traits can be improved simultaneously.

In our study, we proved that MAS for *qhir1* is effective to confirm the true-to-type of haploids. The induced progenies were clustered into two pools according to haplotypes. This allelic clustering can be explained by two hypotheses: (1) single fertilization occurs when only the egg or the central cell is fertilized, resulting in kernels with haploid embryos (Sarkar and Coe, 1966) and (2) selective elimination of inducer genomes from embryonic cells (Zhao et al., 2013). Acknowledging the small sample sizes used, Linnet (1999) suggested that the minimum sample size for optimizing the regression analysis should fall withing the range of 40 to 100 samples. Therefore, conducting further replicated trials with a larger sample size is encouraged before fully realizing the potential of this approach in haploid identification in maize. As a practical proposal, molecular markers could be employed to verify *R1-nj*-based putative haploids at the early seedling stage, not exceeding seven days after planting (DAP). This timeline aligns with the common practice of haploid genome doubling using colchicine at 10-12 DAP (Vanous et al., 2017). To prevent the risk of *R1-nj* marker misclassification, an additional phenotypic marker, the red root phenotype at seedling stage from *Pl-1* gene, was used. This phenotype results from light-independent anthocyanin production, although exposure to light conditions can induce anthocyanin pigmentation for some genotypes (Coe, 1994). Moreover, oil content was used as a screening criterion for haploid and diploid using nuclear magnetic resonance (NMR) (Wang et al., 2016). The success of this method required high-oil haploid inducers (Liu et al., 2022). Preventing high false positives through molecular assays can help to reduce the DH line production costs, because false positives can be discarded prior to haploid genome doubling (Baleroni et al., 2021).

## Conclusions

Our study revealed that implementing marker-assisted selection (MAS) for *qhir1* and *qhir8* at an early generation (F_3_) substantially enhanced the haploid induction rate (HIR) of tropical × temperate haploid inducer families. On average, the HIRs of families homozygous for both *qhir1+* and *qhir8+* were 1–3-fold higher than those homozygous for *qhir1+* only. The *qhir1* marker, utilizing the TaqMan assay, effectively distinguished diploid/haploid progenies at the early seedling stage (≤7 DAP) with high accuracy (100%), as validated by flow cytometric analysis. We propose the integration of MAS to expedite the breeding of haploid inducers for high HIR, complementing the use of the *R1-nj* marker for the identification of true haploids.

## Data availability statement

The datasets presented in this study can be found in online repositories. The names of the repository/repositories and accession number(s) can be found in the article/**Supplementary Material**.

## Author contributions

KK: Writing – original draft, Conceptualization, Data curation, Formal analysis, Methodology. AD: Writing – original draft, Conceptualization, Methodology, Writing – review & editing. KS: Writing – review & editing, Conceptualization, Funding acquisition, Methodology, Supervision. TL: Supervision, Writing – review & editing. SW: Supervision, Writing – review & editing. BT: Supervision, Writing – review & editing. WP: Supervision, Writing – review & editing. TT: Supervision, Writing – review & editing. VR: Conceptualization, Methodology, Supervision, Writing – review & editing. SA: Writing – review & editing, Supervision.
