# Supplementary material for: Accelerating haploid induction rate and haploid validation through marker-assisted selection for qhir1 and qhir8 in maize
Source: Front Plant Sci. 2024 Mar 5;15:1337463. doi: 10.3389/fpls.2024.1337463 (PMC10948437; doi:10.3389/fpls.2024.1337463)
Supplement: Supplementary file 1 [file DataSheet_1.pdf]

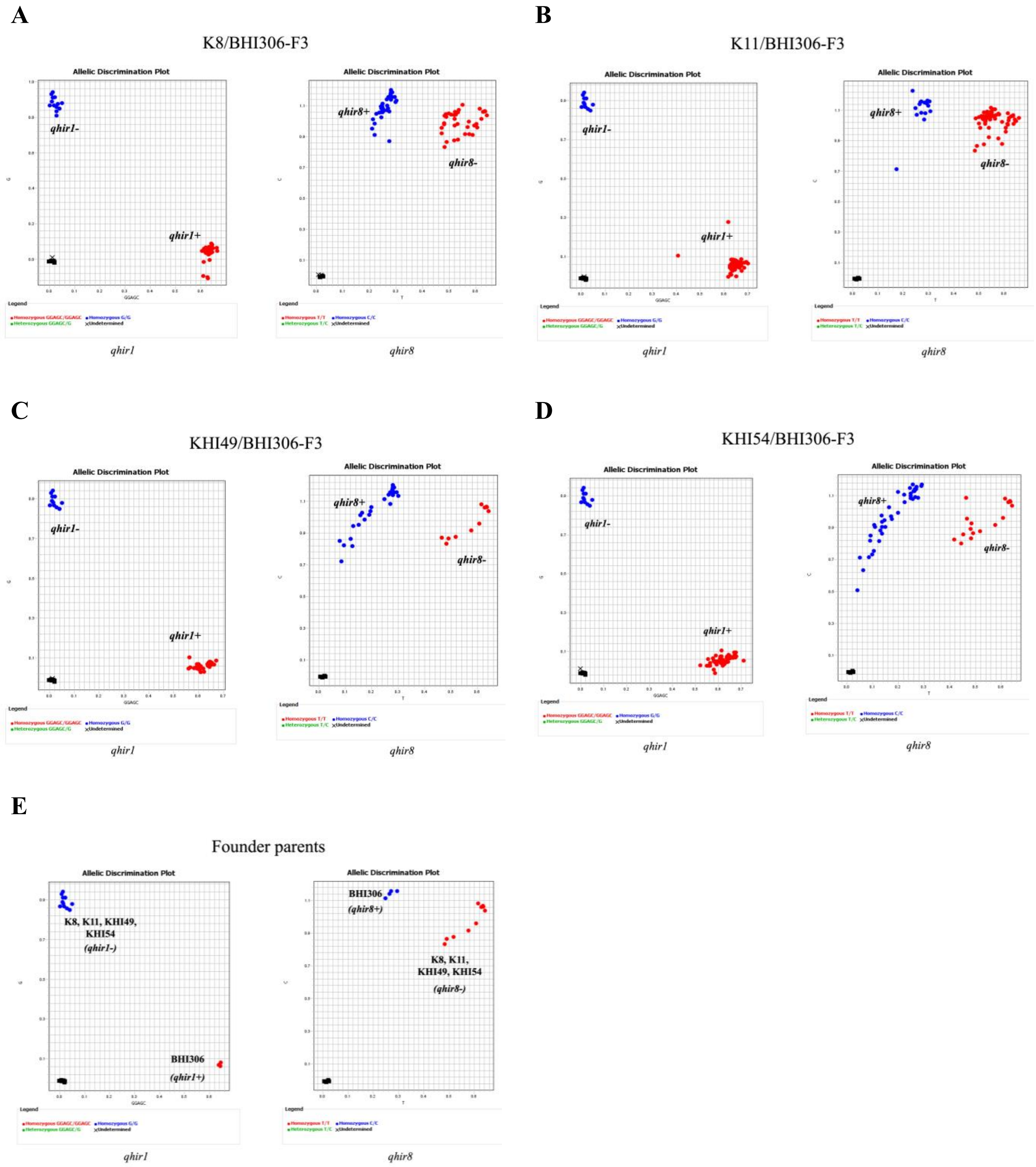

Supplementary Figure S1. The SNP graphs on *qhir1* and *qhir8* markers among individual F_3 haploid inducer plant (A-D) and the founder parents (E). A: 32 F_3 plants of haploid inducer population K8/BHI306; B: 54 F_3 plants of haploid inducer population K11/BHI306; C: 52 F_3 plants of haploid inducer population KHI49/BHI306; D: 99 F_3 plants of haploid inducer

population KHI54/BHI306; E: 14 plants of haploid inducer founder parents. For the *qhir1* marker (left), blue and red dots indicate *qhir1*− and *qhir1*+ genotypes, respectively. For the *qhir8* marker (right), blue and red dots indicate *qhir8*+ and *qhir8*− genotypes, respectively.
