# Supplementary material for: Accelerating haploid induction rate and haploid validation through marker-assisted selection for qhir1 and qhir8 in maize
Source: Front Plant Sci. 2024 Mar 5;15:1337463. doi: 10.3389/fpls.2024.1337463 (PMC10948437; doi:10.3389/fpls.2024.1337463)
Supplement: Supplementary file 2 [file DataSheet_2.pdf]

qhir1

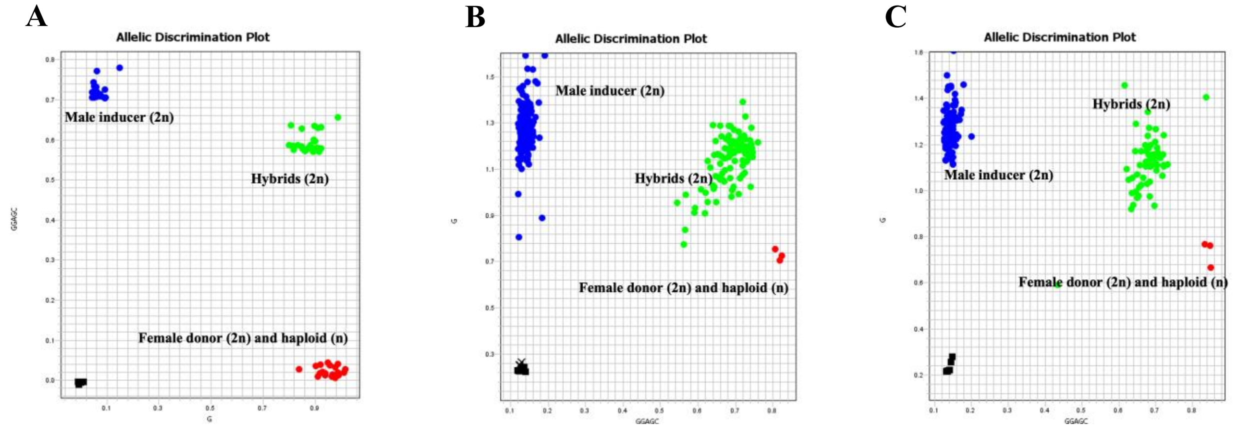

Supplementary Figure S2. The SNP graphs on the *qhir1* marker for ploidy validation of putative haploids and diploids previously derived from the *R1-nj* marker. A: a subset of population P789/BHI306 composed of 11 putative haploids, 10 putative diploids, and 10 samples of BHI306; B: a subset of population P789/(KHI49/BHI306-F_3) composed of 156 putative haploids, 27 putative diploids, and 3 samples of BHI306; C: a subset of population P789/(KHI54/BHI306-F_3) composed of 175 putative haploids, 28 putative diploids, and 3 samples of BHI306. Blue dots indicate genotypes with homozygous GGAGC/GGAGC. Red dots indicate genotypes with homozygous G/G. Green dots indicate genotypes with heterozygous GGAGC/G.

Summary notes

True haploid (n) plants were identified as the genotypes with homozygous G/G because they carried only genomes from the maternal or female donor, whereas true diploid (2n) plants were identified as the genotype with heterozygous GGAGC/G because they carried one copy (GGAGC) from the male inducer and another one copy (G) from the female donor.
