# Supplementary material for: Accelerating haploid induction rate and haploid validation through marker-assisted selection for qhir1 and qhir8 in maize
Source: Front Plant Sci. 2024 Mar 5;15:1337463. doi: 10.3389/fpls.2024.1337463 (PMC10948437; doi:10.3389/fpls.2024.1337463)
Supplement: Supplementary file 3 [file DataSheet_3.pdf]

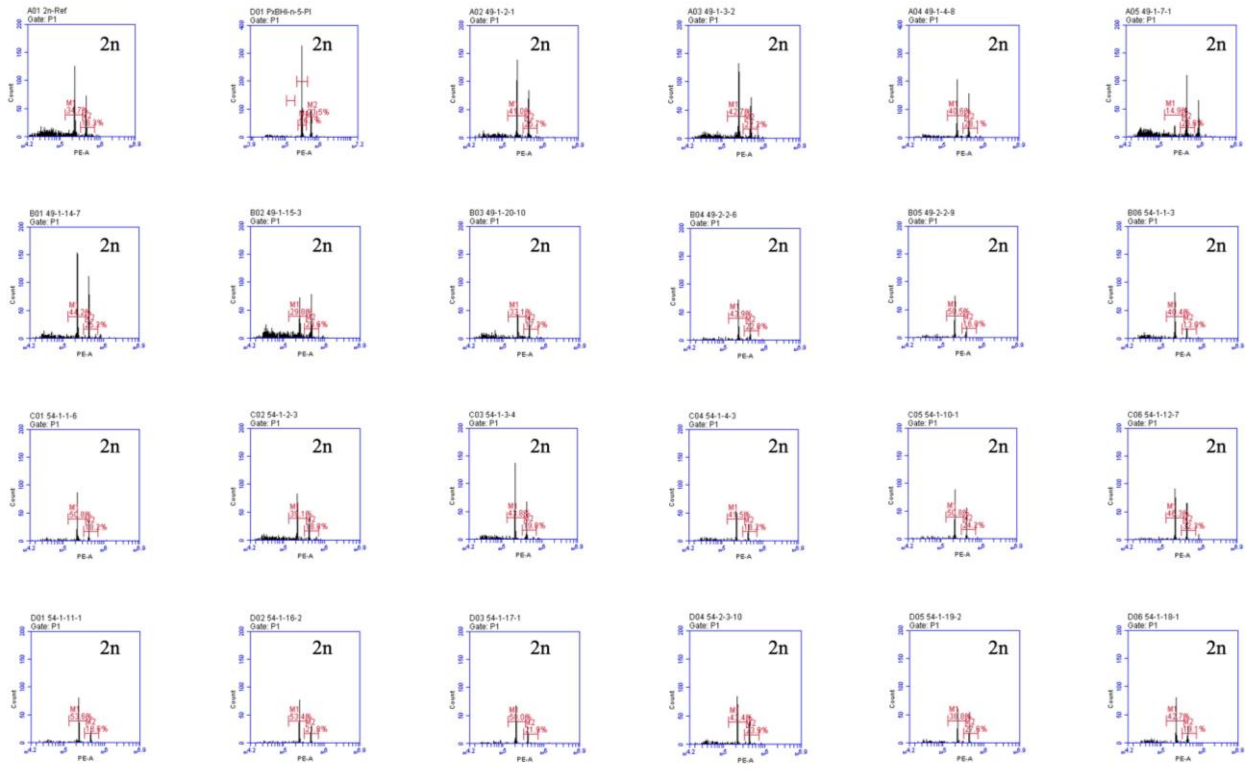

Supplementary Figure S3. Flow cytometry analysis of 24 false positives derived from the *qhir1* marker.

Summary notes

All 24 samples previously classified as putative haploids based on the *R1-nj* marker were found to be true diploids according to the *qhir1* marker. Those 24 false positives were later confirmed as true diploids based on flow cytometry (FC) analysis. According to FC, the first peak (M1) of diploids should surpass the pulse area (PE-A) of 5.0 as the threshold. In contrast, the M1 of haploids should be lower than the PE-A of 5.0 as the threshold. The result of supplementary figure S3 revealed that all samples tested were true diploids (2n).
