# Supplementary material for: Accelerating haploid induction rate and haploid validation through marker-assisted selection for qhir1 and qhir8 in maize
Source: Front Plant Sci. 2024 Mar 5;15:1337463. doi: 10.3389/fpls.2024.1337463 (PMC10948437; doi:10.3389/fpls.2024.1337463)
Supplement: Supplementary file 4 [file DataSheet_4.pdf]

Supplementary Table S1. The genotyping results on *qhir1* and *qhir8* markers and the mean of haploid induction rate (HIR, %) among individual F_3 haploid inducer plant and the founder parents.

Population: K8/BHI306-F3							
**Sample Name**	***qhir1***	***qhir8***	**HIR (%)**	**STDEV**	**Genotype**	**HIR (%)**	**STDEV**
K8/BHI306-F3-53	GGAGC/GGAGC	C/C	4.80	2.6	*qhir1+/qhir8+*	9.36	4.43
K8/BHI306-F3-56	GGAGC/GGAGC	C/C	4.82	2.9			
K8/BHI306-F3-39	GGAGC/GGAGC	C/C	5.87	0.9			
K8/BHI306-F3-61	GGAGC/GGAGC	C/C	6.36	1.2			
K8/BHI306-F3-44	GGAGC/GGAGC	C/C	7.12	2.2			
K8/BHI306-F3-38	GGAGC/GGAGC	C/C	7.47	4.4			
K8/BHI306-F3-18	GGAGC/GGAGC	C/C	7.66	3.1			
K8/BHI306-F3-26	GGAGC/GGAGC	C/C	9.08	2.6			
K8/BHI306-F3-33	GGAGC/GGAGC	C/C	9.11	0.5			
K8/BHI306-F3-21	GGAGC/GGAGC	C/C	9.14	4.5			
K8/BHI306-F3-43	GGAGC/GGAGC	C/C	10.28	3.2			
K8/BHI306-F3-68	GGAGC/GGAGC	C/C	12.90	3.8			
K8/BHI306-F3-54	GGAGC/GGAGC	C/C	15.84	3.2			
K8/BHI306-F3-70	GGAGC/GGAGC	C/C	20.56	4.4			
K8/BHI306-F3-55	GGAGC/GGAGC	T/T	1.44	0.6	*qhir1+/qhir8-*	4.29	2.40
K8/BHI306-F3-52	GGAGC/GGAGC	T/T	1.72	0.9			
K8/BHI306-F3-20	GGAGC/GGAGC	T/T	1.85	1.0			
K8/BHI306-F3-35	GGAGC/GGAGC	T/T	1.91	0.3			
K8/BHI306-F3-50	GGAGC/GGAGC	T/T	2.45	1.0			

K8/BHI306-F3-24	GGAGC/GGAGC	T/T	2.98	2.9			
K8/BHI306-F3-8	GGAGC/GGAGC	T/T	3.01	2.2			
K8/BHI306-F3-51	GGAGC/GGAGC	T/T	3.61	0.6			
K8/BHI306-F3-67	GGAGC/GGAGC	T/T	3.73	1.7			
K8/BHI306-F3-14	GGAGC/GGAGC	T/T	3.82	3.3			
K8/BHI306-F3-71	GGAGC/GGAGC	T/T	3.90	2.7			
K8/BHI306-F3-6	GGAGC/GGAGC	T/T	4.26	0.6			
K8/BHI306-F3-25	GGAGC/GGAGC	T/T	5.14	3.5			
K8/BHI306-F3-27	GGAGC/GGAGC	T/T	5.24	4.2			
K8/BHI306-F3-17	GGAGC/GGAGC	T/T	6.03	4.5			
K8/BHI306-F3-7	GGAGC/GGAGC	T/T	8.38	2.1			
K8/BHI306-F3-29	GGAGC/GGAGC	T/T	8.48	4.4			
K8/BHI306-F3-69	GGAGC/GGAGC	T/T	9.25	3.8			

Population: K11/BHI306-F3

Sample Name	*qhir1*	*qhir8*	HIR (%)	STDEV	Genotype	HIR (%)	STDEV
K11/BHI306-F3-46	GGAGC/GGAGC	C/C	1.89	4.4	*qhir1+/qhir8+*	9.48	5.96
K11/BHI306-F3-43	GGAGC/GGAGC	C/C	2.57	0.9			
K11/BHI306-F3-50	GGAGC/GGAGC	C/C	4.46	3.4			
K11/BHI306-F3-51	GGAGC/GGAGC	C/C	7.05	3.5			
K11/BHI306-F3-19	GGAGC/GGAGC	C/C	7.33	0.9			
K11/BHI306-F3-32	GGAGC/GGAGC	C/C	11.13	2.4			
K11/BHI306-F3-61	GGAGC/GGAGC	C/C	11.47	1.8			
K11/BHI306-F3-10	GGAGC/GGAGC	C/C	13.79	4.5			

K11/BHI306-F3-25	GGAGC/GGAGC	C/C	14.10	2.9			
K11/BHI306-F3-11_2	GGAGC/GGAGC	C/C	20.97	2.5			
K11/BHI306-F3-22	GGAGC/GGAGC	T/T	0.35	0.1	*qhir1+/qhir8-*	4.85	3.39
K11/BHI306-F3-27	GGAGC/GGAGC	T/T	0.52	0.8			
K11/BHI306-F3-38	GGAGC/GGAGC	T/T	1.13	0.5			
K11/BHI306-F3-9	GGAGC/GGAGC	T/T	1.28	1.0			
K11/BHI306-F3-48	GGAGC/GGAGC	T/T	1.43	0.5			
K11/BHI306-F3-26	GGAGC/GGAGC	T/T	1.49	0.4			
K11/BHI306-F3-64	GGAGC/GGAGC	T/T	1.51	0.5			
K11/BHI306-F3-47	GGAGC/GGAGC	T/T	1.62	0.5			
K11/BHI306-F3-42	GGAGC/GGAGC	T/T	1.73	0.7			
K11/BHI306-F3-21	GGAGC/GGAGC	T/T	2.04	1.6			
K11/BHI306-F3-55	GGAGC/GGAGC	T/T	2.16	0.8			
K11/BHI306-F3-41	GGAGC/GGAGC	T/T	2.24	1.7			
K11/BHI306-F3-53	GGAGC/GGAGC	T/T	2.25	0.3			
K11/BHI306-F3-30	GGAGC/GGAGC	T/T	2.43	3.5			
K11/BHI306-F3-36	GGAGC/GGAGC	T/T	2.53	1.0			
K11/BHI306-F3-5	GGAGC/GGAGC	T/T	2.64	1.6			
K11/BHI306-F3-58	GGAGC/GGAGC	T/T	3.40	0.9			
K11/BHI306-F3-29	GGAGC/GGAGC	T/T	3.55	1.1			
K11/BHI306-F3-4	GGAGC/GGAGC	T/T	3.72	2.9			
K11/BHI306-F3-28	GGAGC/GGAGC	T/T	3.74	3.2			
K11/BHI306-F3-54	GGAGC/GGAGC	T/T	3.83	3.0			
K11/BHI306-F3-24	GGAGC/GGAGC	T/T	4.05	1.0			

K11/BHI306-F3-1	GGAGC/GGAGC	T/T	4.32	1.6				
K11/BHI306-F3-3	GGAGC/GGAGC	T/T	4.32	1.5				
K11/BHI306-F3-18	GGAGC/GGAGC	T/T	4.43	1.4				
K11/BHI306-F3-56	GGAGC/GGAGC	T/T	4.49	0.7				
K11/BHI306-F3-17	GGAGC/GGAGC	T/T	4.72	1.6				
K11/BHI306-F3-35	GGAGC/GGAGC	T/T	5.73	2.8				
K11/BHI306-F3-33	GGAGC/GGAGC	T/T	5.76	2.2				
K11/BHI306-F3-57	GGAGC/GGAGC	T/T	5.82	5.2				
K11/BHI306-F3-37	GGAGC/GGAGC	T/T	5.95	1.4				
K11/BHI306-F3-14	GGAGC/GGAGC	T/T	5.99	1.5				
K11/BHI306-F3-68	GGAGC/GGAGC	T/T	6.31	2.0				
K11/BHI306-F3-65	GGAGC/GGAGC	T/T	6.52	4.1				
K11/BHI306-F3-23	GGAGC/GGAGC	T/T	7.03	3.0				
K11/BHI306-F3-13	GGAGC/GGAGC	T/T	5.00	5.0				
K11/BHI306-F3-52	GGAGC/GGAGC	T/T	9.10	2.1				
K11/BHI306-F3-20	GGAGC/GGAGC	T/T	9.34	4.3				
K11/BHI306-F3-12	GGAGC/GGAGC	T/T	9.49	3.2				
K11/BHI306-F3-67	GGAGC/GGAGC	T/T	11.18	4.5				
K11/BHI306-F3-39	GGAGC/GGAGC	T/T	11.38	4.1				
K11/BHI306-F3-31	GGAGC/GGAGC	T/T	11.67	4.4				
K11/BHI306-F3-62	GGAGC/GGAGC	T/T	12.00	2.2				
K11/BHI306-F3-49	GGAGC/GGAGC	T/T	13.04	2.9				

Population: KHI-49/BHI306-F3							
**Sample Name**	***qhir1***	***qhir8***	**HIR (%)**	**STDEV**	**Genotype**	**HIR (%)**	**STDEV**
Ho98-16	GGAGC/GGAGC	C/C	0.00	0.0	*qhir1+/qhir8+*	3.85	2.00
Ho98-6	GGAGC/GGAGC	C/C	1.05	1.1			
Ho98-25	GGAGC/GGAGC	C/C	1.62	2.3			
He105-12	GGAGC/GGAGC	C/C	1.66	0.9			
Ho98-3	GGAGC/GGAGC	C/C	1.89	1.0			
Ho98-2	GGAGC/GGAGC	C/C	2.04	0.6			
He105-17	GGAGC/GGAGC	C/C	2.33	1.1			
He105-14	GGAGC/GGAGC	C/C	2.49	1.1			
Ho98-4	GGAGC/GGAGC	C/C	2.51	0.3			
Ho98-20	GGAGC/GGAGC	C/C	2.61	2.3			
He105-3	GGAGC/GGAGC	C/C	2.86	0.4			
He105-15	GGAGC/GGAGC	C/C	2.91	0.9			
He105-25	GGAGC/GGAGC	C/C	2.98	1.6			
Ho98-23	GGAGC/GGAGC	C/C	2.99	0.9			
Ho98-14	GGAGC/GGAGC	C/C	3.18	1.4			
Ho98-21	GGAGC/GGAGC	C/C	3.18	1.2			
Ho98-5	GGAGC/GGAGC	C/C	3.20	2.2			
Ho98-22	GGAGC/GGAGC	C/C	3.93	2.2			
He105-22	GGAGC/GGAGC	C/C	4.35	2.2			
Ho98-9	GGAGC/GGAGC	C/C	4.88	2.5			
Ho98-7	GGAGC/GGAGC	C/C	4.91	2.2			
Ho98-18	GGAGC/GGAGC	C/C	4.97	0.5			

Ho98-17	GGAGC/GGAGC	C/C	5.13	1.5			
He105-6	GGAGC/GGAGC	C/C	5.48	2.0			
Ho98-24	GGAGC/GGAGC	C/C	5.54	1.8			
He105-21	GGAGC/GGAGC	C/C	5.61	0.6			
Ho98-10	GGAGC/GGAGC	C/C	5.61	2.7			
He105-16	GGAGC/GGAGC	C/C	6.43	2.6			
Ho76-3	GGAGC/GGAGC	C/C	7.26	2.5			
Ho98-1	GGAGC/GGAGC	C/C	7.36	3.3			
Ho98-11	GGAGC/GGAGC	C/C	8.34	3.2			
He105-2	GGAGC/GGAGC	T/T	0.00	0.0	*qhir1+/qhir8-*	1.18	0.95
He105-31	GGAGC/GGAGC	T/T	0.00	0.0			
He105-5	GGAGC/GGAGC	T/T	0.16	0.3			
He105-9	GGAGC/GGAGC	T/T	0.24	0.3			
He105-35	GGAGC/GGAGC	T/T	0.29	0.6			
He105-8	GGAGC/GGAGC	T/T	0.37	0.6			
He105-4	GGAGC/GGAGC	T/T	0.63	0.6			
He105-23	GGAGC/GGAGC	T/T	0.73	0.0			
He105-29	GGAGC/GGAGC	T/T	0.77	0.6			
He105-7	GGAGC/GGAGC	T/T	1.00	1.0			
He105-1	GGAGC/GGAGC	T/T	1.06	0.8			
He105-27	GGAGC/GGAGC	T/T	1.13	1.2			
He105-24	GGAGC/GGAGC	T/T	1.17	0.3			
He105-18	GGAGC/GGAGC	T/T	1.49	0.8			
He105-30	GGAGC/GGAGC	T/T	1.53	0.3			

He105-10	GGAGC/GGAGC	T/T	1.60	1.0			
He105-28	GGAGC/GGAGC	T/T	1.75	0.3			
He105-11	GGAGC/GGAGC	T/T	1.90	0.9			
He105-20	GGAGC/GGAGC	T/T	2.41	0.6			
He105-34	GGAGC/GGAGC	T/T	2.95	2.8			
He105-13	GGAGC/GGAGC	T/T	3.49	0.5			

**Population: KHI-54/BHI306-F3**							
**Sample Name**	***qhir1***	***qhir8***	**HIR (%)**	**STDEV**	**Genotype**	**HIR (%)**	**STDEV**
Ho28-29	GGAGC/GGAGC	C/C	0.61	0.2			
Ho28-28	GGAGC/GGAGC	C/C	1.29	2.1			
He45-17	GGAGC/GGAGC	C/C	1.55	1.1			
Ho28-36	GGAGC/GGAGC	C/C	1.61	1.5			
Ho28-32	GGAGC/GGAGC	C/C	1.62	1.9			
He45-7	GGAGC/GGAGC	C/C	1.69	0.7			
He45-20	GGAGC/GGAGC	C/C	1.80	0.6			
Ho28-18	GGAGC/GGAGC	C/C	1.83	0.6	*qhir1+/qhir8+*	5.61	4.10
Ho28-7	GGAGC/GGAGC	C/C	1.90	1.3			
He45-9	GGAGC/GGAGC	C/C	2.16	1.3			
He45-39	GGAGC/GGAGC	C/C	2.22	1.0			
Ho28-8	GGAGC/GGAGC	C/C	2.37	0.5			
Ho28-34	GGAGC/GGAGC	C/C	2.44	2.7			
Ho28-30	GGAGC/GGAGC	C/C	2.52	1.4			
Ho28-37	GGAGC/GGAGC	C/C	2.54	0.6			

He45-3	GGAGC/GGAGC	C/C	2.84	1.8			
Ho28-9	GGAGC/GGAGC	C/C	3.75	1.9			
Ho28-38	GGAGC/GGAGC	C/C	3.76	2.1			
Ho28-19	GGAGC/GGAGC	C/C	4.16	1.1			
Ho28-40	GGAGC/GGAGC	C/C	4.27	2.2			
Ho28-22	GGAGC/GGAGC	C/C	4.32	3.8			
Ho28-6	GGAGC/GGAGC	C/C	4.63	2.0			
Ho28-25	GGAGC/GGAGC	C/C	4.80	2.5			
Ho28-4	GGAGC/GGAGC	C/C	4.95	4.1			
Ho28-31	GGAGC/GGAGC	C/C	5.28	3.8			
He45-15	GGAGC/GGAGC	C/C	5.32	2.2			
He45-30	GGAGC/GGAGC	C/C	5.83	2.1			
Ho28-27	GGAGC/GGAGC	C/C	6.37	0.2			
Ho28-23	GGAGC/GGAGC	C/C	7.64	4.8			
Ho28-2	GGAGC/GGAGC	C/C	7.79	0.8			
Ho28-3	GGAGC/GGAGC	C/C	7.87	3.2			
Ho28-12	GGAGC/GGAGC	C/C	8.85	1.6			
Ho28-5	GGAGC/GGAGC	C/C	10.23	1.4			
Ho28-16	GGAGC/GGAGC	C/C	10.52	2.1			
Ho28-1	GGAGC/GGAGC	C/C	10.55	4.5			
Ho28-35	GGAGC/GGAGC	C/C	11.58	1.8			
Ho28-14	GGAGC/GGAGC	C/C	11.74	2.7			
Ho28-17	GGAGC/GGAGC	C/C	12.46	2.7			
Ho28-13	GGAGC/GGAGC	C/C	13.67	2.0			

Ho28-15	GGAGC/GGAGC	C/C	13.67	3.9			
Ho28-11	GGAGC/GGAGC	C/C	15.23	4.7			
He96-28	GGAGC/GGAGC	T/T	0.00	0.0	*qhir1+/qhir8-*	1.53	1.60
He96-33	GGAGC/GGAGC	T/T	0.00	0.0			
He96-35	GGAGC/GGAGC	T/T	0.00	0.0			
He96-36	GGAGC/GGAGC	T/T	0.00	0.0			
He96-22	GGAGC/GGAGC	T/T	0.05	0.1			
He96-23	GGAGC/GGAGC	T/T	0.06	0.1			
He96-32	GGAGC/GGAGC	T/T	0.12	0.2			
He96-16	GGAGC/GGAGC	T/T	0.17	0.2			
He96-20	GGAGC/GGAGC	T/T	0.20	0.3			
He96-8	GGAGC/GGAGC	T/T	0.21	0.3			
He96-26	GGAGC/GGAGC	T/T	0.29	0.4			
He96-30	GGAGC/GGAGC	T/T	0.29	0.2			
He45-12	GGAGC/GGAGC	T/T	0.32	0.6			
He96-39	GGAGC/GGAGC	T/T	0.33	0.4			
He45-2	GGAGC/GGAGC	T/T	0.36	0.2			
He96-40	GGAGC/GGAGC	T/T	0.36	0.3			
He96-25	GGAGC/GGAGC	T/T	0.46	0.6			
He96-38	GGAGC/GGAGC	T/T	0.48	0.2			
He96-29	GGAGC/GGAGC	T/T	0.51	0.6			
He96-9	GGAGC/GGAGC	T/T	0.53	0.7			
He96-37	GGAGC/GGAGC	T/T	0.56	0.4			
He45-5	GGAGC/GGAGC	T/T	0.65	1.3			

He96-10	GGAGC/GGAGC	T/T	0.72	0.6				
He45-18	GGAGC/GGAGC	T/T	0.76	0.5				
He96-27	GGAGC/GGAGC	T/T	0.76	0.5				
He45-28	GGAGC/GGAGC	T/T	0.77	0.7				
He45-24	GGAGC/GGAGC	T/T	0.83	0.2				
He96-24	GGAGC/GGAGC	T/T	0.83	0.9				
He96-34	GGAGC/GGAGC	T/T	0.88	1.2				
He96-11	GGAGC/GGAGC	T/T	0.89	0.7				
He45-4	GGAGC/GGAGC	T/T	1.07	0.6				
He45-29	GGAGC/GGAGC	T/T	1.14	0.2				
He45-6	GGAGC/GGAGC	T/T	1.24	0.7				
He45-8	GGAGC/GGAGC	T/T	1.27	0.7				
He45-27	GGAGC/GGAGC	T/T	1.29	0.4				
He45-1	GGAGC/GGAGC	T/T	1.45	0.7				
He45-40	GGAGC/GGAGC	T/T	1.53	0.7				
He45-14	GGAGC/GGAGC	T/T	1.54	1.8				
He45-22	GGAGC/GGAGC	T/T	1.63	1.9				
He45-11	GGAGC/GGAGC	T/T	1.75	1.3				
He45-13	GGAGC/GGAGC	T/T	1.85	1.6				
He45-37	GGAGC/GGAGC	T/T	1.86	2.5				
He45-10	GGAGC/GGAGC	T/T	1.92	0.5				
He96-14	GGAGC/GGAGC	T/T	2.00	1.0				
He45-31	GGAGC/GGAGC	T/T	2.14	0.6				
He45-25	GGAGC/GGAGC	T/T	2.35	0.0				

He45-35	GGAGC/GGAGC	T/T	2.71	0.5			
He45-34	GGAGC/GGAGC	T/T	2.76	2.6			
He45-16	GGAGC/GGAGC	T/T	2.86	2.1			
He96-31	GGAGC/GGAGC	T/T	3.49	3.5			
He96-12	GGAGC/GGAGC	T/T	3.66	2.9			
He45-36	GGAGC/GGAGC	T/T	3.81	1.4			
He45-21	GGAGC/GGAGC	T/T	3.83	1.2			
He45-19	GGAGC/GGAGC	T/T	4.06	1.5			
He45-23	GGAGC/GGAGC	T/T	4.97	1.7			
He45-32	GGAGC/GGAGC	T/T	5.44	4.8			
He45-33	GGAGC/GGAGC	T/T	5.49	2.1			
He96-13	GGAGC/GGAGC	T/T	7.05	2.4			

**Founder parents**					
**Sample Name**	***qhir1***	***qhir8***	**HIR (%)**	**STDEV**	**Genotype**
K8	G/G	T/T	5.40	2.1	*qhir1-/qhir8-*
K11	G/G	T/T	4.70	3.1	*qhir1-/qhir8-*
KHI49	G/G	T/T	3.20	1.2	*qhir1-/qhir8-*
KHI54	G/G	T/T	2.70	2.1	*qhir1-/qhir8-*
BHI306	GGAGC/GGAGC	C/C	10.40	3.5	*qhir1+/qhir8+*

Summary notes

Genotypes were labelled as *qhir1+* if they carry GGAGC/GGAGC, while genotypes were labelled as *qhir8+* if they carry C/C. In population K8/BHI306-F_3, 32 plants were evaluated for *qhir1* and *qhir8* markers, resulting in 14 plants carrying *qhir1+/qhir8+* and 18 plants carrying *qhir1+/qhir8−*. In population K11/BHI306-F_3, 54 plants were evaluated for *qhir1* and *qhir8*

markers, resulting in 10 plants carrying *qhir1+/qhir8+* and 44 plants carrying *qhir1+/qhir8−*. In population KHI49/BHI306-F_3, 52 plants were evaluated for *qhir1* and *qhir8* markers, resulting in 31 plants carrying *qhir1+/qhir8+* and 21 plants carrying *qhir1+/qhir8−*. In population KHI54/BHI306-F_3, 99 plants were evaluated for *qhir1* and *qhir8* markers, resulting in 41 plants carrying *qhir1+/qhir8+* and 58 plants carrying *qhir1+/qhir8−*. While the four female parents, namely K8, K11, KHI49, and KHI54, were *qhir1-/qhir8-*, the male parent BHI306 was *qhir1+/qhir8+*.
