# Supplementary material for: Accelerating haploid induction rate and haploid validation through marker-assisted selection for qhir1 and qhir8 in maize
Source: Front Plant Sci. 2024 Mar 5;15:1337463. doi: 10.3389/fpls.2024.1337463 (PMC10948437; doi:10.3389/fpls.2024.1337463)
Supplement: Supplementary file 5 [file DataSheet_5.pdf]

Supplementary Table S2. Ploidy validation by the *qhir1* marker among putative haploids and diploids derived from the *R1-nj* marker in three populations of induction crosses between a female tester P789 and three male inducers BHI306, KHI49/BHI306-F_3, and KHI54/BHI306-F_3.

Population 1: P789/BHI306-F_1			
**No**	**Sample Name**	***qhir1***	**status**
1	P789/BHI306-F1_n-5	GGAGC/G	false positives (true diploids proven by *qhir1* marker)
2	P789/BHI306-F1_n-1	G/G	true haploids
3	P789/BHI306-F1_n-2	G/G	true haploids
4	P789/BHI306-F1_n-3	G/G	true haploids
5	P789/BHI306-F1_n-4	G/G	true haploids
6	P789/BHI306-F1_n-6	G/G	true haploids
7	P789/BHI306-F1_n-7	G/G	true haploids
8	P789/BHI306-F1_n-8	G/G	true haploids
9	P789/BHI306-F1_n-9	G/G	true haploids
10	P789/BHI306-F1_n-10	G/G	true haploids
11	P789/BHI306-F1_n-11	G/G	true haploids
12	P789/BHI306-F1_2n-1	GGAGC/G	true diploids
13	P789/BHI306-F1_2n-2	GGAGC/G	true diploids
14	P789/BHI306-F1_2n-3	GGAGC/G	true diploids
15	P789/BHI306-F1_2n-4	GGAGC/G	true diploids
16	P789/BHI306-F1_2n-5	GGAGC/G	true diploids
17	P789/BHI306-F1_2n-6	GGAGC/G	true diploids
18	P789/BHI306-F1_2n-7	GGAGC/G	true diploids
19	P789/BHI306-F1_2n-8	GGAGC/G	true diploids
20	P789/BHI306-F1_2n-9	GGAGC/G	true diploids
21	P789/BHI306-F1_2n-10	GGAGC/G	true diploids
22	BHI306	GGAGC/GGAGC	male inducer (2n)
23	BHI306	GGAGC/GGAGC	male inducer (2n)
24	BHI306	GGAGC/GGAGC	male inducer (2n)
25	BHI306	GGAGC/GGAGC	male inducer (2n)
26	BHI306	GGAGC/GGAGC	male inducer (2n)
27	BHI306	GGAGC/GGAGC	male inducer (2n)
28	BHI306	GGAGC/GGAGC	male inducer (2n)
29	BHI306	GGAGC/GGAGC	male inducer (2n)
30	BHI306	GGAGC/GGAGC	male inducer (2n)
31	BHI306	GGAGC/GGAGC	male inducer (2n)

Population 2: P789/(KHI49/BHI306-F_3)-F_1			
**No**	**Sample Name**	***qhir1***	**status**
1	P789/(KHI49/BHI306-F3)-F1_n-1-14-7	GGAGC/G	false positives (true diploids proven by *qhir1* marker)
2	P789/(KHI49/BHI306-F3)-F1_n-1-15-3	GGAGC/G	false positives (true diploids proven by *qhir1* marker)
3	P789/(KHI49/BHI306-F3)-F1_n-1-20-10	GGAGC/G	false positives (true diploids proven by *qhir1* marker)
4	P789/(KHI49/BHI306-F3)-F1_n-1-2-6	GGAGC/G	false positives (true diploids proven by *qhir1* marker)
5	P789/(KHI49/BHI306-F3)-F1_n-1-3-2	GGAGC/G	false positives (true diploids proven by *qhir1* marker)
6	P789/(KHI49/BHI306-F3)-F1_n-1-4-8	GGAGC/G	false positives (true diploids proven by *qhir1* marker)
7	P789/(KHI49/BHI306-F3)-F1_n-1-6-5	GGAGC/G	false positives (true diploids proven by *qhir1* marker)
8	P789/(KHI49/BHI306-F3)-F1_n-1-7-1	GGAGC/G	false positives (true diploids proven by *qhir1* marker)
9	P789/(KHI49/BHI306-F3)-F1_n-2-2-6	GGAGC/G	false positives (true diploids proven by *qhir1* marker)
10	P789/(KHI49/BHI306-F3)-F1_n-2-2-9	GGAGC/G	false positives (true diploids proven by *qhir1* marker)
11	P789/(KHI49/BHI306-F3)-F1_n-1-10-1	G/G	true haploids
12	P789/(KHI49/BHI306-F3)-F1_n-1-10-2	G/G	true haploids
13	P789/(KHI49/BHI306-F3)-F1_n-1-10-3	G/G	true haploids
14	P789/(KHI49/BHI306-F3)-F1_n-1-10-6	G/G	true haploids
15	P789/(KHI49/BHI306-F3)-F1_n-1-10-8	G/G	true haploids
16	P789/(KHI49/BHI306-F3)-F1_n-1-10-9	G/G	true haploids
17	P789/(KHI49/BHI306-F3)-F1_n-1-10-10	G/G	true haploids
18	P789/(KHI49/BHI306-F3)-F1_n-1-11-2	G/G	true haploids
19	P789/(KHI49/BHI306-F3)-F1_n-1-11-3	G/G	true haploids
20	P789/(KHI49/BHI306-F3)-F1_n-1-11-4	G/G	true haploids
21	P789/(KHI49/BHI306-F3)-F1_n-1-11-5	G/G	true haploids
22	P789/(KHI49/BHI306-F3)-F1_n-1-11-6	G/G	true haploids
23	P789/(KHI49/BHI306-F3)-F1_n-1-11-7	G/G	true haploids
24	P789/(KHI49/BHI306-F3)-F1_n-1-11-8	G/G	true haploids
25	P789/(KHI49/BHI306-F3)-F1_n-1-11-9	G/G	true haploids
26	P789/(KHI49/BHI306-F3)-F1_n-1-11-10	G/G	true haploids
27	P789/(KHI49/BHI306-F3)-F1_n-1-1-10	G/G	true haploids
28	P789/(KHI49/BHI306-F3)-F1_n-1-1-1	G/G	true haploids
29	P789/(KHI49/BHI306-F3)-F1_n-1-1-2	G/G	true haploids
30	P789/(KHI49/BHI306-F3)-F1_n-1-12-1	G/G	true haploids
31	P789/(KHI49/BHI306-F3)-F1_n-1-12-10	G/G	true haploids
32	P789/(KHI49/BHI306-F3)-F1_n-1-12-2	G/G	true haploids
33	P789/(KHI49/BHI306-F3)-F1_n-1-12-6	G/G	true haploids
34	P789/(KHI49/BHI306-F3)-F1_n-1-12-7	G/G	true haploids
35	P789/(KHI49/BHI306-F3)-F1_n-1-12-8	G/G	true haploids
36	P789/(KHI49/BHI306-F3)-F1_n-1-1-3	G/G	true haploids
37	P789/(KHI49/BHI306-F3)-F1_n-1-13-1	G/G	true haploids
38	P789/(KHI49/BHI306-F3)-F1_n-1-13-2	G/G	true haploids

39	P789/(KHI49/BHI306-F3)-F1_n-1-13-4	G/G	true haploids
40	P789/(KHI49/BHI306-F3)-F1_n-1-13-5	G/G	true haploids
41	P789/(KHI49/BHI306-F3)-F1_n-1-13-6	G/G	true haploids
42	P789/(KHI49/BHI306-F3)-F1_n-1-13-7	G/G	true haploids
43	P789/(KHI49/BHI306-F3)-F1_n-1-13-8	G/G	true haploids
44	P789/(KHI49/BHI306-F3)-F1_n-1-13-9	G/G	true haploids
45	P789/(KHI49/BHI306-F3)-F1_n-1-1-4	G/G	true haploids
46	P789/(KHI49/BHI306-F3)-F1_n-1-14-1	G/G	true haploids
47	P789/(KHI49/BHI306-F3)-F1_n-1-14-10	G/G	true haploids
48	P789/(KHI49/BHI306-F3)-F1_n-1-14-2	G/G	true haploids
49	P789/(KHI49/BHI306-F3)-F1_n-1-14-4	G/G	true haploids
50	P789/(KHI49/BHI306-F3)-F1_n-1-14-9	G/G	true haploids
51	P789/(KHI49/BHI306-F3)-F1_n-1-1-5	G/G	true haploids
52	P789/(KHI49/BHI306-F3)-F1_n-1-15-1	G/G	true haploids
53	P789/(KHI49/BHI306-F3)-F1_n-1-15-10	G/G	true haploids
54	P789/(KHI49/BHI306-F3)-F1_n-1-15-6	G/G	true haploids
55	P789/(KHI49/BHI306-F3)-F1_n-1-15-7	G/G	true haploids
56	P789/(KHI49/BHI306-F3)-F1_n-1-15-9	G/G	true haploids
57	P789/(KHI49/BHI306-F3)-F1_n-1-1-6	G/G	true haploids
58	P789/(KHI49/BHI306-F3)-F1_n-1-16-3	G/G	true haploids
59	P789/(KHI49/BHI306-F3)-F1_n-1-16-4	G/G	true haploids
60	P789/(KHI49/BHI306-F3)-F1_n-1-16-5	G/G	true haploids
61	P789/(KHI49/BHI306-F3)-F1_n-1-16-7	G/G	true haploids
62	P789/(KHI49/BHI306-F3)-F1_n-1-16-8	G/G	true haploids
63	P789/(KHI49/BHI306-F3)-F1_n-1-1-7	G/G	true haploids
64	P789/(KHI49/BHI306-F3)-F1_n-1-17-1	G/G	true haploids
65	P789/(KHI49/BHI306-F3)-F1_n-1-17-10	G/G	true haploids
66	P789/(KHI49/BHI306-F3)-F1_n-1-17-2	G/G	true haploids
67	P789/(KHI49/BHI306-F3)-F1_n-1-17-6	G/G	true haploids
68	P789/(KHI49/BHI306-F3)-F1_n-1-17-7	G/G	true haploids
69	P789/(KHI49/BHI306-F3)-F1_n-1-17-8	G/G	true haploids
70	P789/(KHI49/BHI306-F3)-F1_n-1-17-9	G/G	true haploids
71	P789/(KHI49/BHI306-F3)-F1_n-1-18-1	G/G	true haploids
72	P789/(KHI49/BHI306-F3)-F1_n-1-18-2	G/G	true haploids
73	P789/(KHI49/BHI306-F3)-F1_n-1-18-5	G/G	true haploids
74	P789/(KHI49/BHI306-F3)-F1_n-1-18-6	G/G	true haploids
75	P789/(KHI49/BHI306-F3)-F1_n-1-18-7	G/G	true haploids
76	P789/(KHI49/BHI306-F3)-F1_n-1-18-9	G/G	true haploids
77	P789/(KHI49/BHI306-F3)-F1_n-1-1-9	G/G	true haploids
78	P789/(KHI49/BHI306-F3)-F1_n-1-19-1	G/G	true haploids

79	P789/(KHI49/BHI306-F3)-F1_n-1-19-2	G/G	true haploids
80	P789/(KHI49/BHI306-F3)-F1_n-1-19-4	G/G	true haploids
81	P789/(KHI49/BHI306-F3)-F1_n-1-19-7	G/G	true haploids
82	P789/(KHI49/BHI306-F3)-F1_n-1-19-8	G/G	true haploids
83	P789/(KHI49/BHI306-F3)-F1_n-1-19-9	G/G	true haploids
84	P789/(KHI49/BHI306-F3)-F1_n-1-20-1	G/G	true haploids
85	P789/(KHI49/BHI306-F3)-F1_n-1-20-2	G/G	true haploids
86	P789/(KHI49/BHI306-F3)-F1_n-1-20-4	G/G	true haploids
87	P789/(KHI49/BHI306-F3)-F1_n-1-20-5	G/G	true haploids
88	P789/(KHI49/BHI306-F3)-F1_n-1-20-7	G/G	true haploids
89	P789/(KHI49/BHI306-F3)-F1_n-1-20-9	G/G	true haploids
90	P789/(KHI49/BHI306-F3)-F1_n-1-2-1	G/G	true haploids
91	P789/(KHI49/BHI306-F3)-F1_n-1-2-10	G/G	true haploids
92	P789/(KHI49/BHI306-F3)-F1_n-1-2-2	G/G	true haploids
93	P789/(KHI49/BHI306-F3)-F1_n-1-2-3	G/G	true haploids
94	P789/(KHI49/BHI306-F3)-F1_n-1-2-4	G/G	true haploids
95	P789/(KHI49/BHI306-F3)-F1_n-1-2-5	G/G	true haploids
96	P789/(KHI49/BHI306-F3)-F1_n-1-2-7	G/G	true haploids
97	P789/(KHI49/BHI306-F3)-F1_n-1-2-8	G/G	true haploids
98	P789/(KHI49/BHI306-F3)-F1_n-1-2-9	G/G	true haploids
99	P789/(KHI49/BHI306-F3)-F1_n-1-3-4	G/G	true haploids
100	P789/(KHI49/BHI306-F3)-F1_n-1-3-5	G/G	true haploids
101	P789/(KHI49/BHI306-F3)-F1_n-1-3-6	G/G	true haploids
102	P789/(KHI49/BHI306-F3)-F1_n-1-3-7	G/G	true haploids
103	P789/(KHI49/BHI306-F3)-F1_n-1-3-8	G/G	true haploids
104	P789/(KHI49/BHI306-F3)-F1_n-1-3-9	G/G	true haploids
105	P789/(KHI49/BHI306-F3)-F1_n-1-4-3	G/G	true haploids
106	P789/(KHI49/BHI306-F3)-F1_n-1-4-4	G/G	true haploids
107	P789/(KHI49/BHI306-F3)-F1_n-1-4-7	G/G	true haploids
108	P789/(KHI49/BHI306-F3)-F1_n-1-5-10	G/G	true haploids
109	P789/(KHI49/BHI306-F3)-F1_n-1-5-2	G/G	true haploids
110	P789/(KHI49/BHI306-F3)-F1_n-1-5-3	G/G	true haploids
111	P789/(KHI49/BHI306-F3)-F1_n-1-5-4	G/G	true haploids
112	P789/(KHI49/BHI306-F3)-F1_n-1-5-5	G/G	true haploids
113	P789/(KHI49/BHI306-F3)-F1_n-1-5-6	G/G	true haploids
114	P789/(KHI49/BHI306-F3)-F1_n-1-5-7	G/G	true haploids
115	P789/(KHI49/BHI306-F3)-F1_n-1-5-8	G/G	true haploids
116	P789/(KHI49/BHI306-F3)-F1_n-1-5-9	G/G	true haploids
117	P789/(KHI49/BHI306-F3)-F1_n-1-6-10	G/G	true haploids
118	P789/(KHI49/BHI306-F3)-F1_n-1-6-2	G/G	true haploids

119	P789/(KHI49/BHI306-F3)-F1_n-1-6-3	G/G	true haploids
120	P789/(KHI49/BHI306-F3)-F1_n-1-6-4	G/G	true haploids
121	P789/(KHI49/BHI306-F3)-F1_n-1-6-6	G/G	true haploids
122	P789/(KHI49/BHI306-F3)-F1_n-1-6-7	G/G	true haploids
123	P789/(KHI49/BHI306-F3)-F1_n-1-6-9	G/G	true haploids
124	P789/(KHI49/BHI306-F3)-F1_n-1-7-2	G/G	true haploids
125	P789/(KHI49/BHI306-F3)-F1_n-1-7-3	G/G	true haploids
126	P789/(KHI49/BHI306-F3)-F1_n-1-7-4	G/G	true haploids
127	P789/(KHI49/BHI306-F3)-F1_n-1-7-5	G/G	true haploids
128	P789/(KHI49/BHI306-F3)-F1_n-1-7-6	G/G	true haploids
129	P789/(KHI49/BHI306-F3)-F1_n-1-7-7	G/G	true haploids
130	P789/(KHI49/BHI306-F3)-F1_n-1-7-8	G/G	true haploids
131	P789/(KHI49/BHI306-F3)-F1_n-1-7-9	G/G	true haploids
132	P789/(KHI49/BHI306-F3)-F1_n-1-8-1	G/G	true haploids
133	P789/(KHI49/BHI306-F3)-F1_n-1-8-3	G/G	true haploids
134	P789/(KHI49/BHI306-F3)-F1_n-1-8-4	G/G	true haploids
135	P789/(KHI49/BHI306-F3)-F1_n-1-8-5	G/G	true haploids
136	P789/(KHI49/BHI306-F3)-F1_n-1-8-6	G/G	true haploids
137	P789/(KHI49/BHI306-F3)-F1_n-1-8-7	G/G	true haploids
138	P789/(KHI49/BHI306-F3)-F1_n-1-8-8	G/G	true haploids
139	P789/(KHI49/BHI306-F3)-F1_n-1-9-1	G/G	true haploids
140	P789/(KHI49/BHI306-F3)-F1_n-1-9-10	G/G	true haploids
141	P789/(KHI49/BHI306-F3)-F1_n-1-9-3	G/G	true haploids
142	P789/(KHI49/BHI306-F3)-F1_n-1-9-4	G/G	true haploids
143	P789/(KHI49/BHI306-F3)-F1_n-1-9-5	G/G	true haploids
144	P789/(KHI49/BHI306-F3)-F1_n-1-9-6	G/G	true haploids
145	P789/(KHI49/BHI306-F3)-F1_n-1-9-7	G/G	true haploids
146	P789/(KHI49/BHI306-F3)-F1_n-1-9-8	G/G	true haploids
147	P789/(KHI49/BHI306-F3)-F1_n-1-9-9	G/G	true haploids
148	P789/(KHI49/BHI306-F3)-F1_n-2-1-3	G/G	true haploids
149	P789/(KHI49/BHI306-F3)-F1_n-2-1-5	G/G	true haploids
150	P789/(KHI49/BHI306-F3)-F1_n-2-1-6	G/G	true haploids
151	P789/(KHI49/BHI306-F3)-F1_n-2-1-7	G/G	true haploids
152	P789/(KHI49/BHI306-F3)-F1_n-2-1-8	G/G	true haploids
153	P789/(KHI49/BHI306-F3)-F1_n-2-2-1	G/G	true haploids
154	P789/(KHI49/BHI306-F3)-F1_n-2-2-4	G/G	true haploids
155	P789/(KHI49/BHI306-F3)-F1_n-2-2-7	G/G	true haploids
156	P789/(KHI49/BHI306-F3)-F1_n-2-2-8	G/G	true haploids
157	P789/(KHI49/BHI306-F3)-F1_2n-2-4-1	GGAGC/G	true diploids
158	P789/(KHI49/BHI306-F3)-F1_2n-2-4-10	GGAGC/G	true diploids

159	P789/(KHI49/BHI306-F3)-F1_2n-2-4-2	GGAGC/G	true diploids
160	P789/(KHI49/BHI306-F3)-F1_2n-2-4-3	GGAGC/G	true diploids
161	P789/(KHI49/BHI306-F3)-F1_2n-2-4-4	GGAGC/G	true diploids
162	P789/(KHI49/BHI306-F3)-F1_2n-2-4-5	GGAGC/G	true diploids
163	P789/(KHI49/BHI306-F3)-F1_2n-2-4-6	GGAGC/G	true diploids
164	P789/(KHI49/BHI306-F3)-F1_2n-2-4-8	GGAGC/G	true diploids
165	P789/(KHI49/BHI306-F3)-F1_2n-2-4-9	GGAGC/G	true diploids
166	P789/(KHI49/BHI306-F3)-F1_2n-2-5-1	GGAGC/G	true diploids
167	P789/(KHI49/BHI306-F3)-F1_2n-2-5-10	GGAGC/G	true diploids
168	P789/(KHI49/BHI306-F3)-F1_2n-2-5-2	GGAGC/G	true diploids
169	P789/(KHI49/BHI306-F3)-F1_2n-2-5-3	GGAGC/G	true diploids
170	P789/(KHI49/BHI306-F3)-F1_2n-2-5-4	GGAGC/G	true diploids
171	P789/(KHI49/BHI306-F3)-F1_2n-2-5-5	GGAGC/G	true diploids
172	P789/(KHI49/BHI306-F3)-F1_2n-2-5-6	GGAGC/G	true diploids
173	P789/(KHI49/BHI306-F3)-F1_2n-2-5-7	GGAGC/G	true diploids
174	P789/(KHI49/BHI306-F3)-F1_2n-2-5-8	GGAGC/G	true diploids
175	P789/(KHI49/BHI306-F3)-F1_2n-2-6-1	GGAGC/G	true diploids
176	P789/(KHI49/BHI306-F3)-F1_2n-2-6-10	GGAGC/G	true diploids
177	P789/(KHI49/BHI306-F3)-F1_2n-2-6-2	GGAGC/G	true diploids
178	P789/(KHI49/BHI306-F3)-F1_2n-2-6-3	GGAGC/G	true diploids
179	P789/(KHI49/BHI306-F3)-F1_2n-2-6-4	GGAGC/G	true diploids
180	P789/(KHI49/BHI306-F3)-F1_2n-2-6-5	GGAGC/G	true diploids
181	P789/(KHI49/BHI306-F3)-F1_2n-2-6-6	GGAGC/G	true diploids
182	P789/(KHI49/BHI306-F3)-F1_2n-2-6-7	GGAGC/G	true diploids
183	P789/(KHI49/BHI306-F3)-F1_2n-2-6-9	GGAGC/G	true diploids
184	BHI306	GGAGC/GGAGC	male inducer (2n)
185	BHI306	GGAGC/GGAGC	male inducer (2n)
186	BHI306	GGAGC/GGAGC	male inducer (2n)

Population 3: P789/(KHI54/BHI306-F_3)-F_1			
**No**	**Sample Name**	***qhir1***	**status**
1	P789/(KHI54/BHI306-F3)-F1_n-1-1-3	GGAGC/G	false positives (true diploids proven by *qhir1* marker)
2	P789/(KHI54/BHI306-F3)-F1_n-1-1-6	GGAGC/G	false positives (true diploids proven by *qhir1* marker)
3	P789/(KHI54/BHI306-F3)-F1_n-1-2-3	GGAGC/G	false positives (true diploids proven by *qhir1* marker)
4	P789/(KHI54/BHI306-F3)-F1_n-1-3-4	GGAGC/G	false positives (true diploids proven by *qhir1* marker)
5	P789/(KHI54/BHI306-F3)-F1_n-1-4-3	GGAGC/G	false positives (true diploids proven by *qhir1* marker)
6	P789/(KHI54/BHI306-F3)-F1_n-1-10-1	GGAGC/G	false positives (true diploids proven by *qhir1* marker)
7	P789/(KHI54/BHI306-F3)-F1_n-1-11-1	GGAGC/G	false positives (true diploids proven by *qhir1* marker)
8	P789/(KHI54/BHI306-F3)-F1_n-1-12-7	GGAGC/G	false positives (true diploids proven by *qhir1* marker)
9	P789/(KHI54/BHI306-F3)-F1_n-1-16-2	GGAGC/G	false positives (true diploids proven by *qhir1* marker)
10	P789/(KHI54/BHI306-F3)-F1_n-1-17-1	GGAGC/G	false positives (true diploids proven by *qhir1* marker)
11	P789/(KHI54/BHI306-F3)-F1_n-1-18-1	GGAGC/G	false positives (true diploids proven by *qhir1* marker)
12	P789/(KHI54/BHI306-F3)-F1_n-1-19-2	GGAGC/G	false positives (true diploids proven by *qhir1* marker)
13	P789/(KHI54/BHI306-F3)-F1_n-2-3-10	GGAGC/G	false positives (true diploids proven by *qhir1* marker)
14	P789/(KHI54/BHI306-F3)-F1_n-1-10-2	G/G	true haploids
15	P789/(KHI54/BHI306-F3)-F1_n-1-10-3	G/G	true haploids
16	P789/(KHI54/BHI306-F3)-F1_n-1-10-4	G/G	true haploids
17	P789/(KHI54/BHI306-F3)-F1_n-1-10-7	G/G	true haploids
18	P789/(KHI54/BHI306-F3)-F1_n-1-1-1	G/G	true haploids
19	P789/(KHI54/BHI306-F3)-F1_n-1-11-3	G/G	true haploids
20	P789/(KHI54/BHI306-F3)-F1_n-1-11-4	G/G	true haploids
21	P789/(KHI54/BHI306-F3)-F1_n-1-11-5	G/G	true haploids
22	P789/(KHI54/BHI306-F3)-F1_n-1-11-6	G/G	true haploids
23	P789/(KHI54/BHI306-F3)-F1_n-1-11-7	G/G	true haploids
24	P789/(KHI54/BHI306-F3)-F1_n-1-11-8	G/G	true haploids
25	P789/(KHI54/BHI306-F3)-F1_n-1-1-2	G/G	true haploids
26	P789/(KHI54/BHI306-F3)-F1_n-1-12-1	G/G	true haploids
27	P789/(KHI54/BHI306-F3)-F1_n-1-12-10	G/G	true haploids
28	P789/(KHI54/BHI306-F3)-F1_n-1-12-2	G/G	true haploids
29	P789/(KHI54/BHI306-F3)-F1_n-1-12-3	G/G	true haploids
30	P789/(KHI54/BHI306-F3)-F1_n-1-12-4	G/G	true haploids
31	P789/(KHI54/BHI306-F3)-F1_n-1-12-9	G/G	true haploids
32	P789/(KHI54/BHI306-F3)-F1_n-1-13-1	G/G	true haploids
33	P789/(KHI54/BHI306-F3)-F1_n-1-13-10	G/G	true haploids
34	P789/(KHI54/BHI306-F3)-F1_n-1-13-4	G/G	true haploids
35	P789/(KHI54/BHI306-F3)-F1_n-1-13-5	G/G	true haploids
36	P789/(KHI54/BHI306-F3)-F1_n-1-13-6	G/G	true haploids
37	P789/(KHI54/BHI306-F3)-F1_n-1-13-7	G/G	true haploids
38	P789/(KHI54/BHI306-F3)-F1_n-1-13-8	G/G	true haploids

39	P789/(KHI54/BHI306-F3)-F1_n-1-13-9	G/G	true haploids
40	P789/(KHI54/BHI306-F3)-F1_n-1-1-4	G/G	true haploids
41	P789/(KHI54/BHI306-F3)-F1_n-1-14-1	G/G	true haploids
42	P789/(KHI54/BHI306-F3)-F1_n-1-14-2	G/G	true haploids
43	P789/(KHI54/BHI306-F3)-F1_n-1-14-3	G/G	true haploids
44	P789/(KHI54/BHI306-F3)-F1_n-1-14-4	G/G	true haploids
45	P789/(KHI54/BHI306-F3)-F1_n-1-14-5	G/G	true haploids
46	P789/(KHI54/BHI306-F3)-F1_n-1-15-1	G/G	true haploids
47	P789/(KHI54/BHI306-F3)-F1_n-1-15-3	G/G	true haploids
48	P789/(KHI54/BHI306-F3)-F1_n-1-15-4	G/G	true haploids
49	P789/(KHI54/BHI306-F3)-F1_n-1-15-5	G/G	true haploids
50	P789/(KHI54/BHI306-F3)-F1_n-1-15-6	G/G	true haploids
51	P789/(KHI54/BHI306-F3)-F1_n-1-15-7	G/G	true haploids
52	P789/(KHI54/BHI306-F3)-F1_n-1-15-8	G/G	true haploids
53	P789/(KHI54/BHI306-F3)-F1_n-1-15-9	G/G	true haploids
54	P789/(KHI54/BHI306-F3)-F1_n-1-16-1	G/G	true haploids
55	P789/(KHI54/BHI306-F3)-F1_n-1-16-10	G/G	true haploids
56	P789/(KHI54/BHI306-F3)-F1_n-1-16-3	G/G	true haploids
57	P789/(KHI54/BHI306-F3)-F1_n-1-16-5	G/G	true haploids
58	P789/(KHI54/BHI306-F3)-F1_n-1-16-8	G/G	true haploids
59	P789/(KHI54/BHI306-F3)-F1_n-1-16-9	G/G	true haploids
60	P789/(KHI54/BHI306-F3)-F1_n-1-17-6	G/G	true haploids
61	P789/(KHI54/BHI306-F3)-F1_n-1-17-7	G/G	true haploids
62	P789/(KHI54/BHI306-F3)-F1_n-1-17-9	G/G	true haploids
63	P789/(KHI54/BHI306-F3)-F1_n-1-18-10	G/G	true haploids
64	P789/(KHI54/BHI306-F3)-F1_n-1-18-2	G/G	true haploids
65	P789/(KHI54/BHI306-F3)-F1_n-1-18-5	G/G	true haploids
66	P789/(KHI54/BHI306-F3)-F1_n-1-18-6	G/G	true haploids
67	P789/(KHI54/BHI306-F3)-F1_n-1-18-9	G/G	true haploids
68	P789/(KHI54/BHI306-F3)-F1_n-1-19-1	G/G	true haploids
69	P789/(KHI54/BHI306-F3)-F1_n-1-19-10	G/G	true haploids
70	P789/(KHI54/BHI306-F3)-F1_n-1-19-5	G/G	true haploids
71	P789/(KHI54/BHI306-F3)-F1_n-1-20-1	G/G	true haploids
72	P789/(KHI54/BHI306-F3)-F1_n-1-20-10	G/G	true haploids
73	P789/(KHI54/BHI306-F3)-F1_n-1-20-2	G/G	true haploids
74	P789/(KHI54/BHI306-F3)-F1_n-1-20-4	G/G	true haploids
75	P789/(KHI54/BHI306-F3)-F1_n-1-20-5	G/G	true haploids
76	P789/(KHI54/BHI306-F3)-F1_n-1-20-7	G/G	true haploids
77	P789/(KHI54/BHI306-F3)-F1_n-1-20-8	G/G	true haploids
78	P789/(KHI54/BHI306-F3)-F1_n-1-2-2	G/G	true haploids

79	P789/(KHI54/BHI306-F3)-F1_n-1-2-4	G/G	true haploids
80	P789/(KHI54/BHI306-F3)-F1_n-1-2-5	G/G	true haploids
81	P789/(KHI54/BHI306-F3)-F1_n-1-2-6	G/G	true haploids
82	P789/(KHI54/BHI306-F3)-F1_n-1-2-7	G/G	true haploids
83	P789/(KHI54/BHI306-F3)-F1_n-1-2-9	G/G	true haploids
84	P789/(KHI54/BHI306-F3)-F1_n-1-3-1	G/G	true haploids
85	P789/(KHI54/BHI306-F3)-F1_n-1-3-10	G/G	true haploids
86	P789/(KHI54/BHI306-F3)-F1_n-1-3-5	G/G	true haploids
87	P789/(KHI54/BHI306-F3)-F1_n-1-3-6	G/G	true haploids
88	P789/(KHI54/BHI306-F3)-F1_n-1-3-7	G/G	true haploids
89	P789/(KHI54/BHI306-F3)-F1_n-1-3-9	G/G	true haploids
90	P789/(KHI54/BHI306-F3)-F1_n-1-4-10	G/G	true haploids
91	P789/(KHI54/BHI306-F3)-F1_n-1-4-2	G/G	true haploids
92	P789/(KHI54/BHI306-F3)-F1_n-1-4-7	G/G	true haploids
93	P789/(KHI54/BHI306-F3)-F1_n-1-5-1	G/G	true haploids
94	P789/(KHI54/BHI306-F3)-F1_n-1-5-10	G/G	true haploids
95	P789/(KHI54/BHI306-F3)-F1_n-1-5-2	G/G	true haploids
96	P789/(KHI54/BHI306-F3)-F1_n-1-5-4	G/G	true haploids
97	P789/(KHI54/BHI306-F3)-F1_n-1-5-7	G/G	true haploids
98	P789/(KHI54/BHI306-F3)-F1_n-1-5-8	G/G	true haploids
99	P789/(KHI54/BHI306-F3)-F1_n-1-5-9	G/G	true haploids
100	P789/(KHI54/BHI306-F3)-F1_n-1-6-1	G/G	true haploids
101	P789/(KHI54/BHI306-F3)-F1_n-1-6-10	G/G	true haploids
102	P789/(KHI54/BHI306-F3)-F1_n-1-6-6	G/G	true haploids
103	P789/(KHI54/BHI306-F3)-F1_n-1-6-8	G/G	true haploids
104	P789/(KHI54/BHI306-F3)-F1_n-1-7-4	G/G	true haploids
105	P789/(KHI54/BHI306-F3)-F1_n-1-7-5	G/G	true haploids
106	P789/(KHI54/BHI306-F3)-F1_n-1-7-6	G/G	true haploids
107	P789/(KHI54/BHI306-F3)-F1_n-1-7-7	G/G	true haploids
108	P789/(KHI54/BHI306-F3)-F1_n-1-7-9	G/G	true haploids
109	P789/(KHI54/BHI306-F3)-F1_n-1-8-10	G/G	true haploids
110	P789/(KHI54/BHI306-F3)-F1_n-1-8-2	G/G	true haploids
111	P789/(KHI54/BHI306-F3)-F1_n-1-8-3	G/G	true haploids
112	P789/(KHI54/BHI306-F3)-F1_n-1-8-4	G/G	true haploids
113	P789/(KHI54/BHI306-F3)-F1_n-1-8-6	G/G	true haploids
114	P789/(KHI54/BHI306-F3)-F1_n-1-8-8	G/G	true haploids
115	P789/(KHI54/BHI306-F3)-F1_n-1-8-9	G/G	true haploids
116	P789/(KHI54/BHI306-F3)-F1_n-1-9-10	G/G	true haploids
117	P789/(KHI54/BHI306-F3)-F1_n-1-9-2	G/G	true haploids
118	P789/(KHI54/BHI306-F3)-F1_n-1-9-3	G/G	true haploids

119	P789/(KHI54/BHI306-F3)-F1_n-1-9-4	G/G	true haploids
120	P789/(KHI54/BHI306-F3)-F1_n-1-9-5	G/G	true haploids
121	P789/(KHI54/BHI306-F3)-F1_n-1-9-6	G/G	true haploids
122	P789/(KHI54/BHI306-F3)-F1_n-1-9-8	G/G	true haploids
123	P789/(KHI54/BHI306-F3)-F1_n-1-9-9	G/G	true haploids
124	P789/(KHI54/BHI306-F3)-F1_n-2-1-1	G/G	true haploids
125	P789/(KHI54/BHI306-F3)-F1_n-2-1-3	G/G	true haploids
126	P789/(KHI54/BHI306-F3)-F1_n-2-1-4	G/G	true haploids
127	P789/(KHI54/BHI306-F3)-F1_n-2-1-5	G/G	true haploids
128	P789/(KHI54/BHI306-F3)-F1_n-2-1-8	G/G	true haploids
129	P789/(KHI54/BHI306-F3)-F1_n-2-2-10	G/G	true haploids
130	P789/(KHI54/BHI306-F3)-F1_n-2-2-2	G/G	true haploids
131	P789/(KHI54/BHI306-F3)-F1_n-2-2-6	G/G	true haploids
132	P789/(KHI54/BHI306-F3)-F1_n-2-2-7	G/G	true haploids
133	P789/(KHI54/BHI306-F3)-F1_n-2-2-9	G/G	true haploids
134	P789/(KHI54/BHI306-F3)-F1_n-2-3-1	G/G	true haploids
135	P789/(KHI54/BHI306-F3)-F1_n-2-3-2	G/G	true haploids
136	P789/(KHI54/BHI306-F3)-F1_n-2-3-4	G/G	true haploids
137	P789/(KHI54/BHI306-F3)-F1_n-2-3-5	G/G	true haploids
138	P789/(KHI54/BHI306-F3)-F1_n-2-3-6	G/G	true haploids
139	P789/(KHI54/BHI306-F3)-F1_n-2-3-7	G/G	true haploids
140	P789/(KHI54/BHI306-F3)-F1_n-2-4-1	G/G	true haploids
141	P789/(KHI54/BHI306-F3)-F1_n-2-4-10	G/G	true haploids
142	P789/(KHI54/BHI306-F3)-F1_n-2-4-2	G/G	true haploids
143	P789/(KHI54/BHI306-F3)-F1_n-2-4-3	G/G	true haploids
144	P789/(KHI54/BHI306-F3)-F1_n-2-4-4	G/G	true haploids
145	P789/(KHI54/BHI306-F3)-F1_n-2-4-5	G/G	true haploids
146	P789/(KHI54/BHI306-F3)-F1_n-2-4-6	G/G	true haploids
147	P789/(KHI54/BHI306-F3)-F1_n-2-4-7	G/G	true haploids
148	P789/(KHI54/BHI306-F3)-F1_n-2-4-9	G/G	true haploids
149	P789/(KHI54/BHI306-F3)-F1_n-2-5-1	G/G	true haploids
150	P789/(KHI54/BHI306-F3)-F1_n-2-5-3	G/G	true haploids
151	P789/(KHI54/BHI306-F3)-F1_n-2-5-4	G/G	true haploids
152	P789/(KHI54/BHI306-F3)-F1_n-2-5-5	G/G	true haploids
153	P789/(KHI54/BHI306-F3)-F1_n-2-5-6	G/G	true haploids
154	P789/(KHI54/BHI306-F3)-F1_n-2-5-7	G/G	true haploids
155	P789/(KHI54/BHI306-F3)-F1_n-2-5-8	G/G	true haploids
156	P789/(KHI54/BHI306-F3)-F1_n-2-5-9	G/G	true haploids
157	P789/(KHI54/BHI306-F3)-F1_n-2-6-1	G/G	true haploids
158	P789/(KHI54/BHI306-F3)-F1_n-2-6-10	G/G	true haploids

159	P789/(KHI54/BHI306-F3)-F1_n-2-6-2	G/G	true haploids
160	P789/(KHI54/BHI306-F3)-F1_n-2-6-3	G/G	true haploids
161	P789/(KHI54/BHI306-F3)-F1_n-2-6-5	G/G	true haploids
162	P789/(KHI54/BHI306-F3)-F1_n-2-6-6	G/G	true haploids
163	P789/(KHI54/BHI306-F3)-F1_n-2-6-7	G/G	true haploids
164	P789/(KHI54/BHI306-F3)-F1_n-2-6-8	G/G	true haploids
165	P789/(KHI54/BHI306-F3)-F1_n-2-6-9	G/G	true haploids
166	P789/(KHI54/BHI306-F3)-F1_n-2-7-1	G/G	true haploids
167	P789/(KHI54/BHI306-F3)-F1_n-2-7-10	G/G	true haploids
168	P789/(KHI54/BHI306-F3)-F1_n-2-7-3	G/G	true haploids
169	P789/(KHI54/BHI306-F3)-F1_n-2-7-5	G/G	true haploids
170	P789/(KHI54/BHI306-F3)-F1_n-2-7-6	G/G	true haploids
171	P789/(KHI54/BHI306-F3)-F1_n-2-7-8	G/G	true haploids
172	P789/(KHI54/BHI306-F3)-F1_n-2-7-9	G/G	true haploids
173	P789/(KHI54/BHI306-F3)-F1_n-2-8-1	G/G	true haploids
174	P789/(KHI54/BHI306-F3)-F1_n-2-8-2	G/G	true haploids
175	P789/(KHI54/BHI306-F3)-F1_n-2-8-3	G/G	true haploids
176	P789/(KHI54/BHI306-F3)-F1_2n-2-10-1	GGAGC/G	true diploids
177	P789/(KHI54/BHI306-F3)-F1_2n-2-10-10	GGAGC/G	true diploids
178	P789/(KHI54/BHI306-F3)-F1_2n-2-10-2	GGAGC/G	true diploids
179	P789/(KHI54/BHI306-F3)-F1_2n-2-10-3	GGAGC/G	true diploids
180	P789/(KHI54/BHI306-F3)-F1_2n-2-10-4	GGAGC/G	true diploids
181	P789/(KHI54/BHI306-F3)-F1_2n-2-10-5	GGAGC/G	true diploids
182	P789/(KHI54/BHI306-F3)-F1_2n-2-10-6	GGAGC/G	true diploids
183	P789/(KHI54/BHI306-F3)-F1_2n-2-10-7	GGAGC/G	true diploids
184	P789/(KHI54/BHI306-F3)-F1_2n-2-10-8	GGAGC/G	true diploids
185	P789/(KHI54/BHI306-F3)-F1_2n-2-10-9	GGAGC/G	true diploids
186	P789/(KHI54/BHI306-F3)-F1_2n-2-11-1	GGAGC/G	true diploids
187	P789/(KHI54/BHI306-F3)-F1_2n-2-11-10	GGAGC/G	true diploids
188	P789/(KHI54/BHI306-F3)-F1_2n-2-11-2	GGAGC/G	true diploids
189	P789/(KHI54/BHI306-F3)-F1_2n-2-11-3	GGAGC/G	true diploids
190	P789/(KHI54/BHI306-F3)-F1_2n-2-11-4	GGAGC/G	true diploids
191	P789/(KHI54/BHI306-F3)-F1_2n-2-11-5	GGAGC/G	true diploids
192	P789/(KHI54/BHI306-F3)-F1_2n-2-11-6	GGAGC/G	true diploids
193	P789/(KHI54/BHI306-F3)-F1_2n-2-11-7	GGAGC/G	true diploids
194	P789/(KHI54/BHI306-F3)-F1_2n-2-11-8	GGAGC/G	true diploids
195	P789/(KHI54/BHI306-F3)-F1_2n-2-11-9	GGAGC/G	true diploids
196	P789/(KHI54/BHI306-F3)-F1_2n-2-12-1	GGAGC/G	true diploids
197	P789/(KHI54/BHI306-F3)-F1_2n-2-12-10	GGAGC/G	true diploids
198	P789/(KHI54/BHI306-F3)-F1_2n-2-12-2	GGAGC/G	true diploids

199	P789/(KHI54/BHI306-F3)-F1_2n-2-12-3	GGAGC/G	true diploids
200	P789/(KHI54/BHI306-F3)-F1_2n-2-12-6	GGAGC/G	true diploids
201	P789/(KHI54/BHI306-F3)-F1_2n-2-12-7	GGAGC/G	true diploids
202	P789/(KHI54/BHI306-F3)-F1_2n-2-12-8	GGAGC/G	true diploids
203	P789/(KHI54/BHI306-F3)-F1_2n-2-12-9	GGAGC/G	true diploids
204	BHI306	GGAGC/GGAGC	male inducer (2n)
205	BHI306	GGAGC/GGAGC	male inducer (2n)
206	BHI306	GGAGC/GGAGC	male inducer (2n)

Summary notes

Plant samples with GGAGC/G (*qhir1+/qhir1−*) are defined as true diploids. Plant samples with G/G (*qhir1−*) are defined as true haploids. In population 1, P789/BHI306-F_1, 1 of 11 putative haploids was confirmed as true diploids, indicating there was a false positive. In population 2, P789/(KHI49/BHI306-F_3)-F_1, 10 of 156 putative haploids were confirmed as true diploids, indicating there were 10 false positives. In population 3, P789/(KHI54/BHI306-F_3)-F_1, 13 of 175 putative haploids were confirmed as true diploids, indicating there were 13 false positives. In all three populations, all putative diploids were confirmed as true diploids.
